# Reversal of *C9orf72* mutation-induced transcriptional dysregulation and pathology in cultured human neurons by allele-specific excision

**DOI:** 10.1073/pnas.2307814121

**Published:** 2024-04-15

**Authors:** Aradhana Sachdev, Kamaljot Gill, Maria Sckaff, Alisha M. Birk, Olubankole Aladesuyi Arogundade, Katherine A. Brown, Runvir S. Chouhan, Patrick Oliver Issagholian-Lewin, Esha Patel, Hannah L. Watry, Mylinh T. Bernardi, Kathleen C. Keough, Yu-Chih Tsai, Alec Simon Tulloch Smith, Bruce R. Conklin, Claire Dudley Clelland

**Affiliations:** ^a^Gladstone Institutes, San Francisco, CA 94158; ^b^Weill Institute for Neurosciences, University of California San Francisco, San Francisco, CA 94158; ^c^Memory & Aging Center, Department of Neurology, University of California San Francisco, San Francisco, CA 94158; ^d^Pacific Biosciences, Menlo Park, CA 94025; ^e^Department of Physiology and Biophysics, University of Washington, Seattle, WA 98195; ^f^The Institute for Stem Cell and Regenerative Medicine, University of Washington, Seattle, WA 98195; ^g^Department of Medicine, University of California San Francisco, San Francisco, CA 94143; ^h^Department of Ophthalmology, University of California San Francisco, San Francisco, CA 94143; ^i^Department of Pharmacology, University of California San Francisco, San Francisco, CA 94158

**Keywords:** C9orf72, ALS, FTD, CRISPR, neurodegeneration

## Abstract

Efforts to genetically reverse C9orf72 pathology have been hampered by our incomplete understanding of the regulation of this complex locus. We generated five different genomic excisions at the *C9orf72* locus in a patient-derived induced pluripotent stem cell (iPSC) line and a non-diseased wild-type (WT) line (11 total isogenic lines), and examined gene expression and pathological hallmarks of C9 frontotemporal dementia/amyotrophic lateral sclerosis in motor neurons differentiated from these lines. Comparing the excisions in these isogenic series removed the confounding effects of different genomic backgrounds and allowed us to probe the effects of specific genomic changes. A coding single nucleotide polymorphism in the patient cell line allowed us to distinguish transcripts from the normal vs. mutant allele. Using digital droplet PCR (ddPCR), we determined that transcription from the mutant allele is upregulated at least 10-fold, and that sense transcription is independently regulated from each allele. Surprisingly, excision of the WT allele increased pathologic dipeptide repeat poly-GP expression from the mutant allele. Importantly, a single allele was sufficient to supply a normal amount of protein, suggesting that the C9orf72 gene is haplo-sufficient in induced motor neurons. Excision of the mutant repeat expansion reverted all pathology (RNA abnormalities, dipeptide repeat production, and TDP-43 pathology) and improved electrophysiological function, whereas silencing sense expression did not eliminate all dipeptide repeat proteins, presumably because of the antisense expression. These data increase our understanding of C9orf72 gene regulation and inform gene therapy approaches, including antisense oligonucleotides (ASOs) and CRISPR gene editing.

## Significance

Here, we provide insight into gene regulation at the *C9orf72* locus that will directly impact the development of therapeutics for *C9orf72*-related diseases including frontotemporal dementia (FTD) and ALS (amyotrophic lateral sclerosis). First, we demonstrate that messenger RNAs (mRNAs) from the mutant *C9orf72* allele, which harbor a large expansion of a six-nucleotide repeat, are selectively upregulated and can be correctly spliced (i.e., rid of the toxic repeat). Second, we show that motor neurons tolerate the loss of one *C9orf72* allele and that removal of the repeat expansion corrects all of the pathological hallmarks of C9-FTD/ALS in cultured motor neurons. Overall our findings support excision of the mutant *C9orf72* allele as a promising therapeutic CRISPR gene editing strategy.

Heterozygous expansion of a GGGGCC repeat in the first intron of the *C9orf72* gene is the most frequent known genetic cause of both frontotemporal dementia (FTD) and amyotrophic lateral sclerosis (ALS) (1–3) (C9-FTD/ALS). The *C9orf72* gene is complex: It has two alternative start sites and can be transcribed in both sense and antisense directions (1, 4). The repeat expansion lies between the two start sites, and is transcribed in both directions. This complexity has made it difficult to understand how the repeat expansion causes pathology, and hampered the design of effective gene-based therapy for C9-FTD/ALS.

The prevalent hypothesis is that C9-FTD/ALS pathology results from toxic products derived from expression of the *C9orf72* repeat expansion itself. Indeed, sense or antisense transcripts that harbor a large repeat expansion produce toxic dipeptide repeat proteins (DPRs) through repeat-associated noncanonical (RAN) translation (4–13), and may disrupt RNA processing by sequestering RNA-binding proteins (9, 14–17). An alternative hypothesis is that the repeat expansion disrupts the sense transcription or translation of the mutant allele’s coding region and causes haplo-insufficiency (18–21). However, haplo-insufficiency is unlikely to be the major contributor to C9-FTD/ALS. The most compelling evidence against this hypothesis is that large-scale population sequencing (such as gnomAD) and clinical sequencing show that people with *C9orf72* heterozygous loss-of-function mutations do not develop C9-FTD/ALS (22). Second, homozygous knock-out mouse models have an autoimmune phenotype but no neurologic disease (23–26). Nevertheless, loss of *C9orf72* function may exacerbate the toxic gain-of-function (25, 27) caused by the repeat expansion. Disentangling these possibilities has been challenging because it has been hard to reliably detect *C9orf72* RNA and protein products and to measure allele-specific function. In addition, traditional sequencing methods are inadequate to accurately size large repeat expansions and confirm that a genomic edit has eliminated it. As a result, experiments often compare cells from a disease-carrier to cells from an unrelated healthy donor, rather than to edited isogenic cells, which is problematic because genetic background can have a big effect on phenotypic outcomes.

To overcome these obstacles, we have recently developed a series of analytical tools (28) that we deployed in the present study. Our goal was to understand the effects of manipulating the *C9orf72* locus on gene expression and on pathology at the cellular level. This type of proof-of-concept study is critical to the development of gene therapies, including gene editing and RNA targeting approaches. We engineered a series of excisions that targeted either the mutant (expanded) allele, the normal (unexpanded) allele, or both alleles in the same patient line. The resulting six isogenic lines allowed us to compare the effects of the genetic manipulations without the confounding effect of varying genetic background. We replicated the isogenic series (five additional cell lines) in a nondiseased control cell line to study the effects of the genetic changes on normal gene function. Each line was made clonal and only harbors one change to the genome.

We carried out these manipulations in human iPSCs and examined their impact on both the RNA and protein products of the *C9orf72* locus, and on a cellular marker of C9/ALS pathology, TDP43, in motor neurons derived from the iPSCs. Our analysis reveals unexpected aspects of gene expression and regulation at the *C9orf72* locus that should inform future therapeutic gene targeting approaches.

## Results

### Engineering 11 Isogenic iPSC Lines across Two Genetic Backgrounds.

**Fig. 1.**
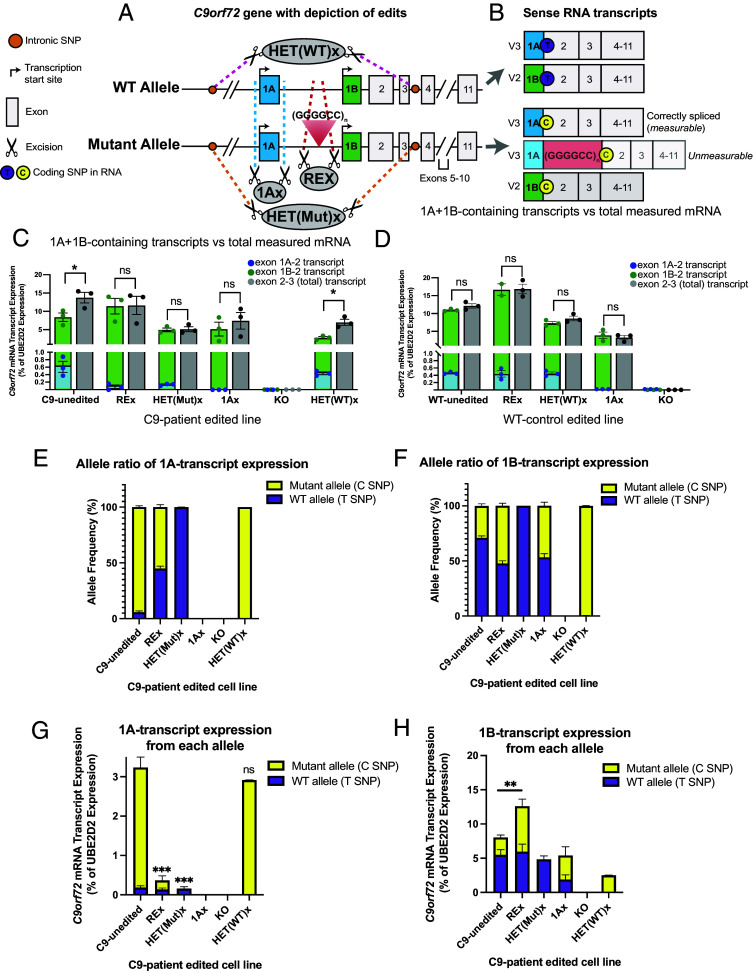
1A-containing transcripts are upregulated from the mutant, but not WT, allele and sense expression is independently controlled by each allele. (*A*) The repeat region of the *C9orf72* gene lies in the 5′ UTR, between alternative start sites that give rise to noncoding exons 1A (blue) and 1B (green). Translation starts in exon 2. Only the allele with the repeat expansion causes disease. We created a series of isogenic lines using CRISPR. REx: excision of the repeat region (~1,057 bp on the mutant allele). 1Ax: 227 bp biallelic excision of exon 1A. HET(Mut)x: 21 kb excision of the mutant allele made by targeting SNPs (orange circles) in cis with the mutation. HET(WT)x: 22 kb excision of the WT allele only. KO was made via biallelic excision starting 5′ to exon 1A and extending through exon 3 (21 to 22 kb; C9-patient line) or exon 2 (7 kb, WT-control line). The schematics of KO lines are shown in SF5 and SF10 (*B*) Schematic of expected 1A and 1B allele-specific transcripts. ddPCR probes were designed to distinguish mRNA transcripts starting at exon 1A (V3) vs. exon 1B (V2), and total *C9orf72* mRNAs (probe spanning exons 2 to 3). A coding SNP allows the quantification of RNA from the WT allele (carrying a T, purple) vs. the mutant allele (carrying a C, yellow). Exon 1A normally splices onto exon 2, but the repeat expansion disrupts this splicing event. Exon 1A transcripts from the mutant (C-allele) are only detected if correctly spliced, due to amplification failure of the repeat region. (*C* and *D*) ddPCR quantification of exon 1A-containing RNA (blue), exon 1B-containing RNA (green), and total *C9orf72* RNA (gray) in isogenic lines from a C9-patient (*C*) or WT control (*D*), normalized to expression of the *UBE2D2* housekeeping gene. In all cell lines, the majority of *C9orf72* mRNAs start at exon 1B, and mRNAs starting at exon 1A are 20 to 100 times less abundant than those starting at exon 1B. In lines harboring a repeat expansion (C9-unedited, HET(WT)x), the sum of exon-1A- + exon-1B-containing transcripts was significantly smaller than the amount of total transcripts (paired *t* test corrected for multiple tests, *P < 0.01). We hypothesize that this gap corresponds to improperly spliced 1A RNA from the mutant allele, since the presence of a repeat expansion would disrupt amplification between exon 1A and exon 2 as well as probe binding. (*E* and *F*) Proportion of 1A (*E*) and 1B (*F*) transcripts coming from the C vs. T allele in unedited and edited C9 lines, as determined by ddPCR. (*G* and *H*) Amounts of 1A (*G*) and 1B (*H*) transcripts relative to *UBE2D2* transcripts in unedited and edited C9 lines, as determined by ddPCR. The mutant allele produces 10× more 1A transcripts (C9-unedited) than WT allele does. Excising the repeat expansion (REx) decreased expression of the 1A transcript from the mutant allele (*G*; Sidak’s multiple comparisons, *P < 0.0001) and increased expression of the 1B transcript from the mutant allele (*H*; Sidak’s multiple comparisons, **P < 0.01) without altering expression of either the 1A (Sidak’s multiple comparisons, *P* > 0.99) or 1B transcript from the WT allele (Sidak’s multiple comparisons, *P* > 0.99). Sense transcription of either allele is independently regulated, as expression of 1A- and 1B- transcripts from one allele was not altered by excision of the other allele (*G*, Sidak’s multiple comparison 1A-T-allele HET(Mut)x vs. C9-unedited *P* > 0.99; 1A-C-allele HET(Mut)x vs. C9-unedited *P* = 0.90; *H*, 1B-T-allele HET(Mut)x vs. C9-unedited *P* = 0.78; 1B-C-allele HET(Mut)x vs. C9-unedited *P* > 0.99). Excision of exon 1A eliminated expression of 1A-transcripts (*G*) and decreased expression of 1B transcripts from the WT (*H*: 1Ax, Sidak’s multiple comparison *P* < 0.05) but not mutant (*H*: 1Ax, Sidak’s multiple comparison *P* = 0.98) alleles. Homozygous KO eliminated both the 1A and 1B transcripts as expected (*G* and *H*). *P < 0.01, **P < 0.001, ***P < 0.0001. Error bars = SEM.

The *C9orf72* repeat region lies between two alternative start sites (1, 2), one in exon 1A, the other in exon 1B (Fig. 1*A*). Our patient line contains ~250 GGGGCC repeats (28) on the mutant allele, and two on the wild-type (WT) allele. The donor was a 37-y-old female patient of European descent who was asymptomatic at the time of donation. We chose this line because of a rare but advantageous single nucleotide polymorphism (SNP) in exon 2 that allows us to quantify gene expression from each allele independently (Fig. 1*B*). From here on out, we refer to this line as C9-unedited. Our control cell line was donated by a healthy 30-y-old subject of mixed European and Asian descent. It contains 10 repeats on one allele and two on the other and is referred to as WT-control.

Before carrying out our edits, we engineered our C9 and WT iPSC lines to contain the hNIL transgene cassette with a TET-on system in the CLYBL safe-harbor locus (29, 30) (*SI Appendix*, Fig. S1). This well-characterized inducible system (29, 30) puts expression of three human transcription factors, NGN2, ISL1, and LHX3, under doxycycline control, which drives the iPSCs to differentiate into motor neurons. The neurons express high levels of motor neuron markers (HB9 and ChAT) compared to iPSCs at the time point investigated in our expression studies (2 wk of age) (*SI Appendix*, Fig. S1 *E* and *F*).

We then introduced Cas9 and gRNA pairs via electroporation into the WT and patient hNIL iPSC lines to generate a variety of excisions of the *C9orf72* genomic locus. We used CRISPOR (31) or AlleleAnalyzer (32) to design gRNAs (*SI Appendix*, Table S1) with the fewest computationally predicted overall off-targets. The genetic change was confirmed by PCR and Sanger sequencing or single-molecule sequencing in sorted single cells or hand-picked clones. All clones had a normal karyotype (*SI Appendix*, Figs. S1–S10).

We first excised the noncoding region containing the repeat expansion (REx, Fig. 1*A* and *SI Appendix*, Fig. S2). Since gRNAs targeting the repeated motif have numerous predicted off-targets throughout the genome, it is not safe to cut within the repeat region itself; instead, we made cuts just 5′ and 3′ to the repeats (Fig. 1*A* REx, *SI Appendix*, Fig. S2). These repeat-flanking sequences are highly conserved and do not offer allele-specific gRNA binding sites. Therefore, this excision was expected to be biallelic. However, by chance it only occurred on the mutant allele in the clone we selected from our patient cell line (*SI Appendix*, Fig. S2), leaving intact the WT allele with its two native repeats. Our WT-control clone with the same edit was biallelic as expected (*SI Appendix*, Fig. S7).

Newer versions of Cas9 can distinguish between alleles that differ by a single nucleotide (33), which allowed us to use SNPs in cis with the mutation to specifically excise the mutant allele. We phased SNPs to the repeat expansion mutation in our patient line using single-molecule sequencing (28). We used AlleleAnalyzer (32) to design allele-specific gRNA pairs based on common heterozygous polymorphisms found in a reference dataset of over 2,500 human genomes from around the world (34). Using two allele-specific gRNAs, we excised 22.2 kb of the mutant allele in the patient line starting 12.3 kb upstream of exon 1A and stretching all the way through exon 3 (Fig. 1*A* HET(Mut)x, *SI Appendix*, Fig. S3). In addition, we made the equivalent 21 kb excision on the WT allele in both the patient and WT lines (Fig. 1*A* HET(WT)x, *SI Appendix*, Figs. S6 and S8) (35).

Our fourth edit, excision of exon 1A, was designed to determine the impact of this exon on *C9orf72* expression and to test the hypothesis that silencing the mutation would suffice to eliminate C9orf72 pathology. Exon 1A includes a transcriptional start site and controls the expression of the *C9orf72* sense-transcript harboring the mutation. We excised it on both alleles (1Ax) in both the patient (Fig. 1 1Ax, *SI Appendix*, Fig. S4) and WT-control lines (*SI Appendix*, Fig. S9). As additional controls, we also created homozygous knock-outs of the gene in our patient and WT lines, using biallelic excisions starting 21 kb upstream of exon 1A and ending in exon 3 (patient line; *SI Appendix*, Fig. S5) or 7 kb upstream of exon 1A and ending in exon 2 (WT line; *SI Appendix*, Fig. S10). We found no detectable off-targets in the edited clonal iPSC lines REx, HET(Mut)x, 1Ax by comparing Sanger sequencing across computationally predicted potential off-target sites (*SI Appendix*, Fig. S11).

### Exon 1A-Transcription Is Upregulated on the Mutant Allele.

We evaluated the effect of each of the edits on *C9orf72* RNA expression in induced motor neurons derived from the edited and unedited iPSC lines. The *C9orf72* locus is known to produce at least three sense mRNAs: variant 1 (exon 1A-short through exon 5), variant 2 (exon 1B-exon 11), and variant 3 (exon 1A-long through exon 11) (1). Using ddPCR probes spanning either the exon 1A-exon 2 or exon 1B-exon 2 splice junctions, we quantified the two major splice forms of *C9orf72*, variant 3 and variant 2, which we refer to as 1A-transcript and 1B-transcript from here on (Fig. 1*B* and *SI Appendix*, Table S2). We were not able to detect variant 1 in our lines, consistent with its low to undetectable expression in human tissue (36, 37). We also quantified total *C9orf72* mRNA using a probe targeting the exon 2-exon 3 junction.

Across all lines, most *C9orf72* mRNAs contained exon 1B, while exon 1A-containing transcripts represented only a small proportion of total transcripts (Fig. 1 *C* and *D*). However, the sum of 1A and 1B transcripts was inferior to the total amount of *C9orf72* transcripts in lines containing the repeat expansion (Fig. 1*C*; C9-unedited, HET(WT)x). This discrepancy was not observed in patient lines in which the mutation was excised (REx, HET(Mut)x) or silenced (1Ax) (Fig. 1*C*) or in any of the WT lines (Fig. 1*D*). We hypothesize the gap corresponds to sense 1A-transcripts that retain the repeat expansion instead of splicing it out; such transcripts cannot be detected by ddPCR, because the repeat expansion disrupts the binding of the ddPCR exon-exon spanning probe and puts too much distance between the primers’ binding sites for a successful PCR. If this hypothesis is correct, the actual proportion of sense 1A-transcripts could reach 30% or more of total *C9orf72* transcripts in lines with the repeat expansion, up from <1% in corrected and WT motor neurons (Fig. 1 *C* and *D*). We report the amount of sense mutant transcript in human induced motor neurons. This estimate does not include antisense repeat-containing transcripts, which are known to occur but are not captured by our assay, and would further increase the total amount of repeat-containing transcripts. We cannot confirm this amount directly, because it is not yet possible to PCR or sequence transcripts harboring a large repeat expansion.

Interestingly, the number of 1A-transcripts (blue bar) decreased in the C9 lines after removal of the repeat region (REx) or excision of the mutant allele (HET(Mut)x) but not after excision of the WT allele (HET(WT)x) (Fig. 1*C*). The removal of the repeat expansion in the patient line (C9-REx and C9-HET(Mut)x) decreased the 1A transcript level below that of unedited WT (0.2 vs. 0.5% of UBE2D2 expression, respectively; Fig. 1*C* vs. Fig. 1*D*). We are cautious to compare expression from the two lines directly because differences in genetic background can influence baseline transcriptional levels. However, we wondered whether editing affected the methylation state which could influence transcriptional regulation. We compared methylation patterns (38) between C9-unedited and the C9-REx and C9-HET(Mut)x iPSCs. We found no differences in methylation between either lines within a 5 kb region around the repeat expansion and including exon 1A and 1B (*SI Appendix*, Fig. S12). In summary, these data suggest that 1A-transcription is upregulated in the diseased state, most probably from the mutant allele.

To test this hypothesis, we determined the proportion of transcripts coming from the WT vs. mutant alleles. We took advantage of a coding SNP (rs10757668) in the exon 2 splice acceptor of our patient line and phased it to the repeat expansion by single-molecule sequencing. Then, using ddPCR probes that targeted either variant of this SNP, we determined the fraction of 1A- and 1B-transcripts derived from each *C9orf72* allele (Fig. 1 *E* and *F*) as well as the amount of 1A and 1B transcripts produced by each allele relative to a house-keeping transcript (Fig. 1 *G* and *H*). Once again and as expected, our assay only detected correctly spliced RNA transcripts and not those retaining the repeat expansion. Nevertheless, most (94%) of the exon 1A-containing transcripts we were able to amplify came from the mutant rather than the WT allele in the unedited patient line (Fig. 1 *E* and *G*, C9-unedited). The imbalance was corrected by repeat expansion excision, which reduced expression from the mutant allele without altering expression from the WT allele (Fig. 1 *E* and *G*, REx). These findings suggest that at least some of the mutant 1A-transcripts can undergo normal splicing. Furthermore, since the mutant and WT 1A transcripts we detect differ only at the SNP (Fig. 1*B*), the excess of mutant 1A transcripts in the unedited C9 line must reflect increased transcription from the mutant allele, rather than a difference in RNA stability. According to our quantification, 1A is at least 10-fold more active in the mutant than the WT allele (Fig. 1*E*, C9-unedited). This is likely an underestimate since we cannot currently measure 1A-transcripts retaining the repeat expansion.

In contrast to 1A transcripts, 1B transcripts came predominantly (>68%) from the WT allele in C9-unedited motor neurons (Fig. 1 *F* and *H*). The balance between the two alleles was fully restored by the allele-specific excision of the repeat expansion (REx), suggesting that the repeat expansion partially inhibits 1B-transcription on the mutant allele. Biallelic excision of exon 1A also restored equal production of 1B transcripts by the two alleles (Fig. 1*F*; 1Ax). However, it reduced expression from the WT allele (Fig. 1*G*; compare height of the purple bar (WT) between 1Ax and C9-unedited), suggesting a positive interaction between 1A and 1B on the WT allele.

### Sense Transcription Is Controlled by Each Allele Independently.

Interestingly, removal of the repeat expansion did not alter the production of 1A and 1B transcripts by the WT allele, and neither did excision of the mutant allele (Fig. 1 *G* and *H*; compare the height of purple bars between C9-unedited, REx and HET(Mut)x). Similarly, removal of the WT allele did not alter expression from the mutant allele (Fig. 1 *G* and *H*, compare the height of yellow bars in HET(WT)x relative to C9-unedited). Thus, expression from either allele is independently regulated. As expected, excision of either allele resulted in elimination of any detectable transcript from that allele (HET(Mut)x and HET(WT)x, Fig. 1 *E* and *H*).

In total, these findings demonstrate that transcripts harboring the repeat expansion can be correctly spliced (i.e., rid of the toxic repeat), an exciting finding that could inspire an additional therapeutic avenue. Second, the repeat expansion increases the activity of the 1A start site and decreases the activity of the 1B start site on the mutant allele but not the WT allele. Finally, sense transcription is allele-independent as removal of either allele did not alter sense expression from the other allele.

### Patient-Derived Lines Lacking Either Allele Produce Normal Levels of Full-Length C9orf72 Protein.

**Fig. 2.**
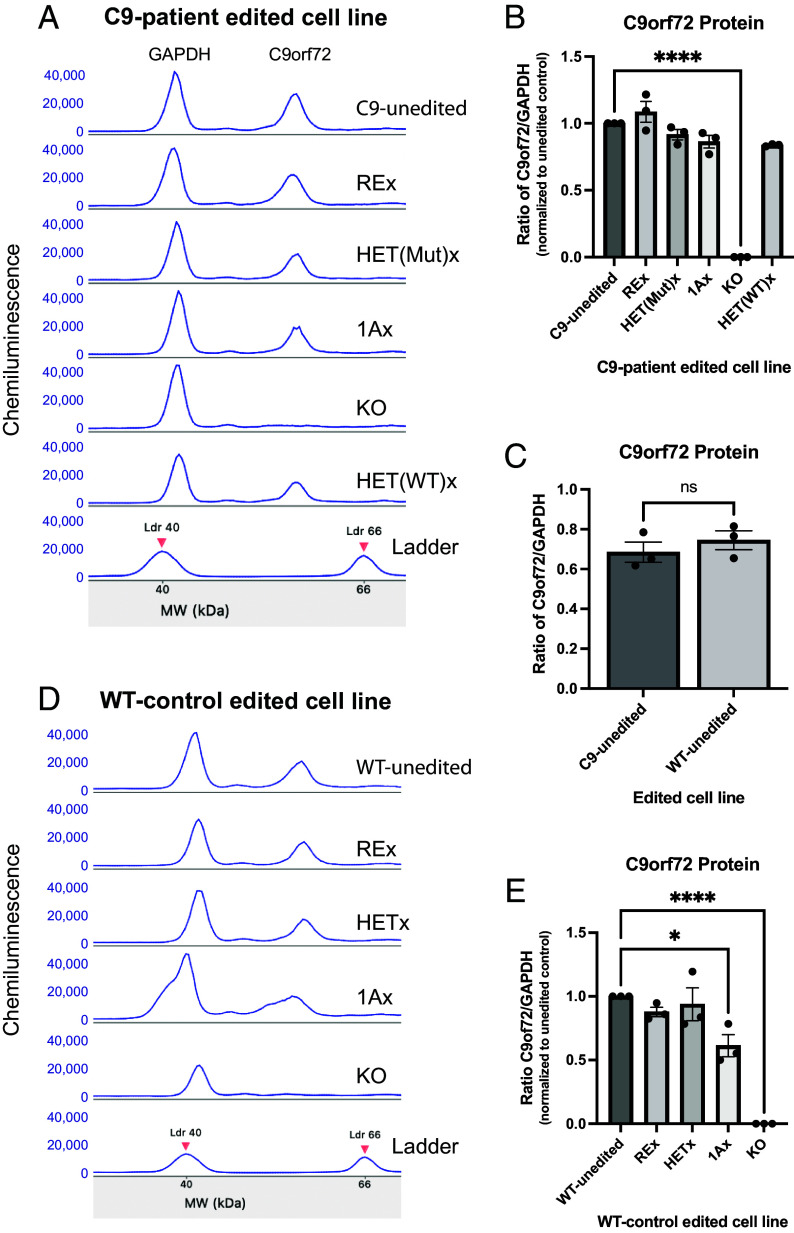
C9orf72 protein abundance is not affected by excision of the repeat expansion or excision of either mutant or WT allele. Quantification of C9orf72 protein expression by WES relative to GAPDH in neurons derived from unedited and edited C9 (*A–C*) and WT (*D* and *E*) iPSCs 2 wk post induction of neuronal differentiation. C9orf72 protein levels were not affected by edits, except for biallelic 1A excision, which reduced protein levels in WT neurons, or homozygous gene KO, which abolished protein signal in both C9 and WT neurons. (1-way ANOVA: C9: F(5,12) = 94.81, *P* < 0.0001; WT: F(4,10) = 32.98, *P* < 0.0001; Dunnett’s multiple comparison test *P < 0.05, ****P < 0.0001). (*C*) There was no significant difference in the amount of C9orf72 protein produced from either the unedited WT or C9-patient line (*P* = 0.43; two-tailed *t* test). Error bars = SEM.

To quantify C9orf72 protein, we used the Simple Western system (WES) after validating antibody specificity using our knock-out line (39). None of the edits in the patient line reduced the C9orf72 protein levels in induced motor neurons (Fig. 2 *A* and *C*). In WT motor neurons, only exon 1A excision reduced C9orf72 expression (Fig. 2 *B* and *D*). Furthermore, unedited WT and C9-patient motor neurons produced equivalent amounts of C9orf72 proteins (Fig. 2*E*). These results indicate that the repeat expansion itself or large excisions of the *C9orf72* locus (such as removal of an entire allele) do not greatly alter total protein levels in motor neurons.

### Removal of the Repeat Expansion Is Required to Eliminate Toxic DPRs.

**Fig. 3.**
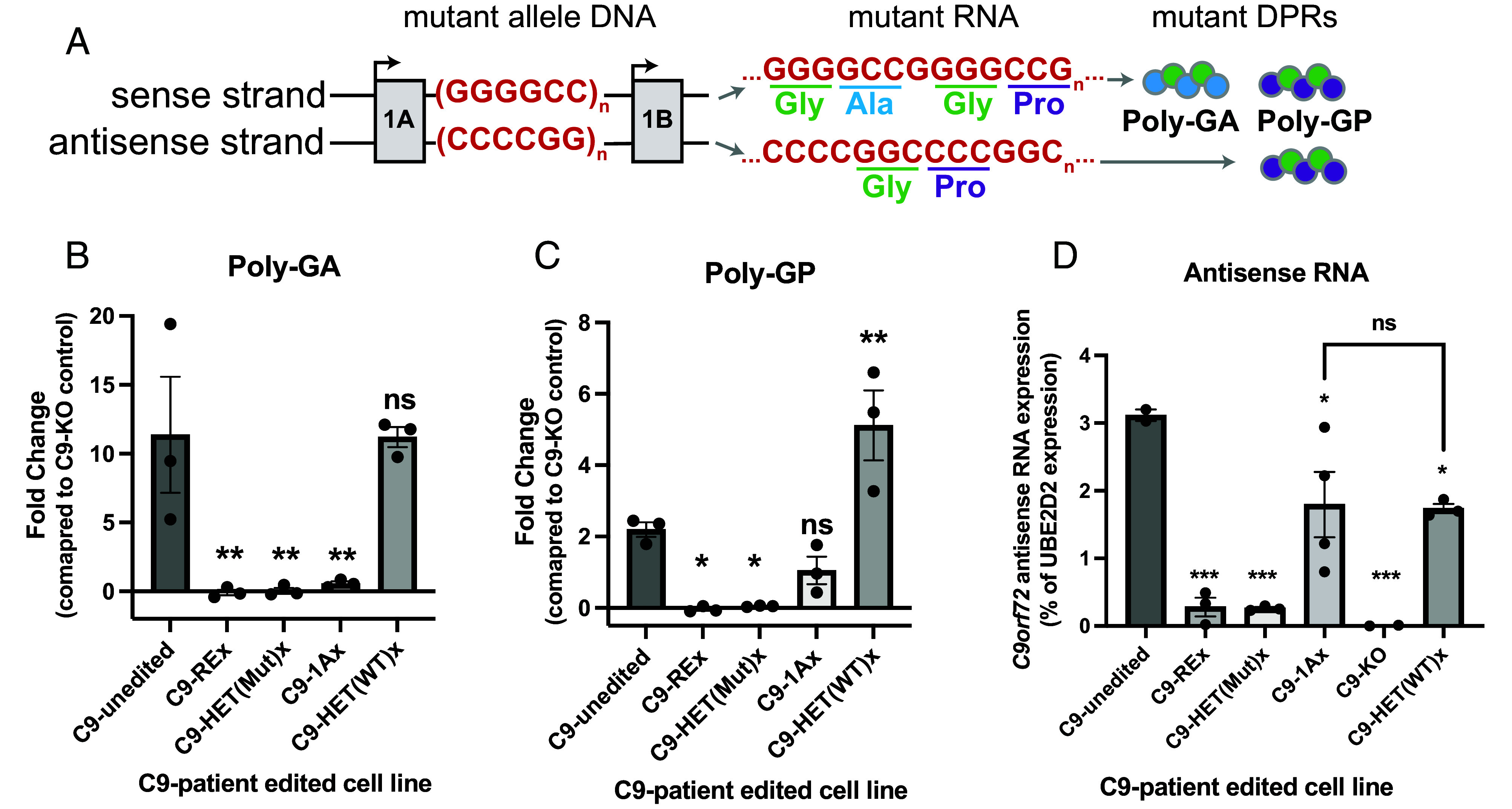
DPRs from sense RNA are eliminated by removal or silencing of the repeat expansion but antisense DPRs are only eliminated by removing the repeat expansion. (*A*) The repeat expansion is transcribed from the sense strand (starting at exon 1A) and the antisense strand (start site unknown) and gives rise to poly-GA and poly-GP peptides through noncanonical repeat-associated non-AUG (RAN) translation. Only sense transcription can give rise to a Poly-GA peptide, whereas Poly-GP can arise from the sense or antisense strands. (*B* and *C*) Quantification of Poly-GA (*B*) and Poly-GP (*C*) in neurons derived from unedited and edited C9 cell lines, relative to a baseline noise established by the C9 KO line, as measured via MSD ELISA. We measured DPRs in neurons 2 wk postinduction. Only excision of the repeat expansion or the mutant allele abolishes production of both Poly-GA (1-way ANOVA F(4,10) = 10.12, *P* < 0.001) and Poly-GP peptides (1-way ANOVA F(4,10) = 19.66, *P* < 0.0001). Excision of exon 1A only abolishes expression of Poly-GA, consistent with silencing of sense but not anti-sense transcription. Excision of the WT allele (HET(WTx)) more than doubled expression of Poly-GP. (*D*) Antisense RNA transcript measured by ddPCR is elevated in edited cell lines that retain the repeat expansion (C9-1Ax, C9-HET(WT)x) (1-way ANOVA F(5,11) = 13.07, *P* < 0.001). *P < 0.5, **P < 0.01 by Dunnett’s or Tukey’s multiple comparisons test sample vs. C9-unedited unless otherwise indicated by comparison bracket. Error bars = SEM.

*C9orf72* is transcribed off of both the sense and anti-sense strands, in both normal and diseased cells (Fig. 3*A*) (7, 15, 40, 41). Our data suggest that sense transcription of the repeat region starts from exon 1A, since excision of exon 1A closed the gap in “undetectable” sense transcript (Fig. 1*C*). However, it is unresolved where anti-sense transcription initiates. Regardless, both sense and anti-sense transcripts harboring the repeat expansion are translated through noncanonical RAN translation to form five DPRs thought to be toxic (6, 7, 10). These DPRs are likely to be variable in size. They deposit in the brains of C9orf72 mutation carriers but not in nondiseased controls and are not found in other neurodegenerative conditions (i.e., DPRs are a specific C9-pathology) (4, 42). The absence of DPRs in WT cells may reflect the rapid splicing out of the repeat region from sense 1A transcripts, the inefficiency of noncanonical RAN translation when there are too few repeats, or a combination of both.

We tested whether removing the repeat region could abrogate DPR production. We evaluated ten antibodies targeting each of these DPRs using Meso Scale Discovery’s (MSD) enzyme-linked immunosorbent assay (ELISA) immunoassay. We found two antibody combinations that could reliably detect the presence of poly-GA and poly-GP DPRs above the background level defined by our knock-out (KO) line (28, 39). The other antibodies had comparable signal in the KO line and the unedited C9-patient line, suggesting they were not specific to the *C9orf72* DPRs. Using these two combinations, we measured poly-GA and poly-GP DPRs in 2-wk-old neurons derived from our C9-isogenic series.

The poly-GA is encoded exclusively on the sense strand, whereas the poly-GP can come from either strand (Fig. 3*A*). As expected, Poly-GA expression was eliminated by removal of the REx and excision of the mutant allele (HET(Mut)x), or by elimination of sense transcription through excision of exon 1A (1Ax) (Fig. 3*B*). Also as expected, all poly-GP was eliminated by removal of the repeat expansion or mutant allele. However, some poly-GP remained after excision of exon 1A (Fig. 3*C*, 1Ax). Since we know that sense 1A transcript is eliminated in the 1Ax line (Fig. 1 *C*, *E*, and *G*), the poly-GP we detect in this line must arise from antisense mutant transcript. We quantify DPR (poly-GP) expression specifically from the anti-sense transcript in patient-derived motor neurons rather than in artificial expression systems. Our data indicate that at least one-third to one-half of poly-GP derives from antisense transcription, and confirm that eliminating toxic DPRs requires the removal of the expanded repeat region. In total, these data also indicate that removal of the repeat expansion, rather than silencing sense expression, is required to completely eliminate DPRs.

### Excision of the WT Allele Increased Poly-GP Expression.

A surprising finding is that excision of the WT allele more than doubled the amount of poly-GP expressed from the mutant allele (Fig. 3*C*, HET(WT)x). By contrast, poly-GA expression was not changed by excision of the WT allele (Fig. 3*B*, HET(WT)x), consistent with our earlier observation that sense transcription from one allele was unaffected by removal of the other allele (Figs. 1 *E*–*H* and 3*B*). Because poly-GP is translated from both sense and antisense transcripts, we asked whether an increase in antisense transcription from the loss of the WT allele could account for the increase in poly-GP expression in the HET(WT)x induced motor neuron. We measured intron-containing (antisense) RNA using ddPCR across the edited C9-induced motor neurons. Removal of the WT allele lowered antisense transcription by one-third in C9-HET(WT)x compared to C9-unedited (Fig. 3*D*). Additionally, elimination of sense transcription by excising exon 1A also lowered antisense transcription by one-third and was not statistically different than removal of the WT allele (Fig. 3*D*, 1Ax vs. HET(WT)x). Altogether, these findings suggest that the upregulation of poly-GP in HET(WT)x induced motor neurons (Fig. 3*C*) is not driven by an increase in either the sense or antisense transcripts we measured.

### Biallelic and Allele-Specific Excisions Revert TDP-43 Pathology in 7-wk-old Motor Neurons Derived from Edited Patient iPSCs.

**Fig. 4.**
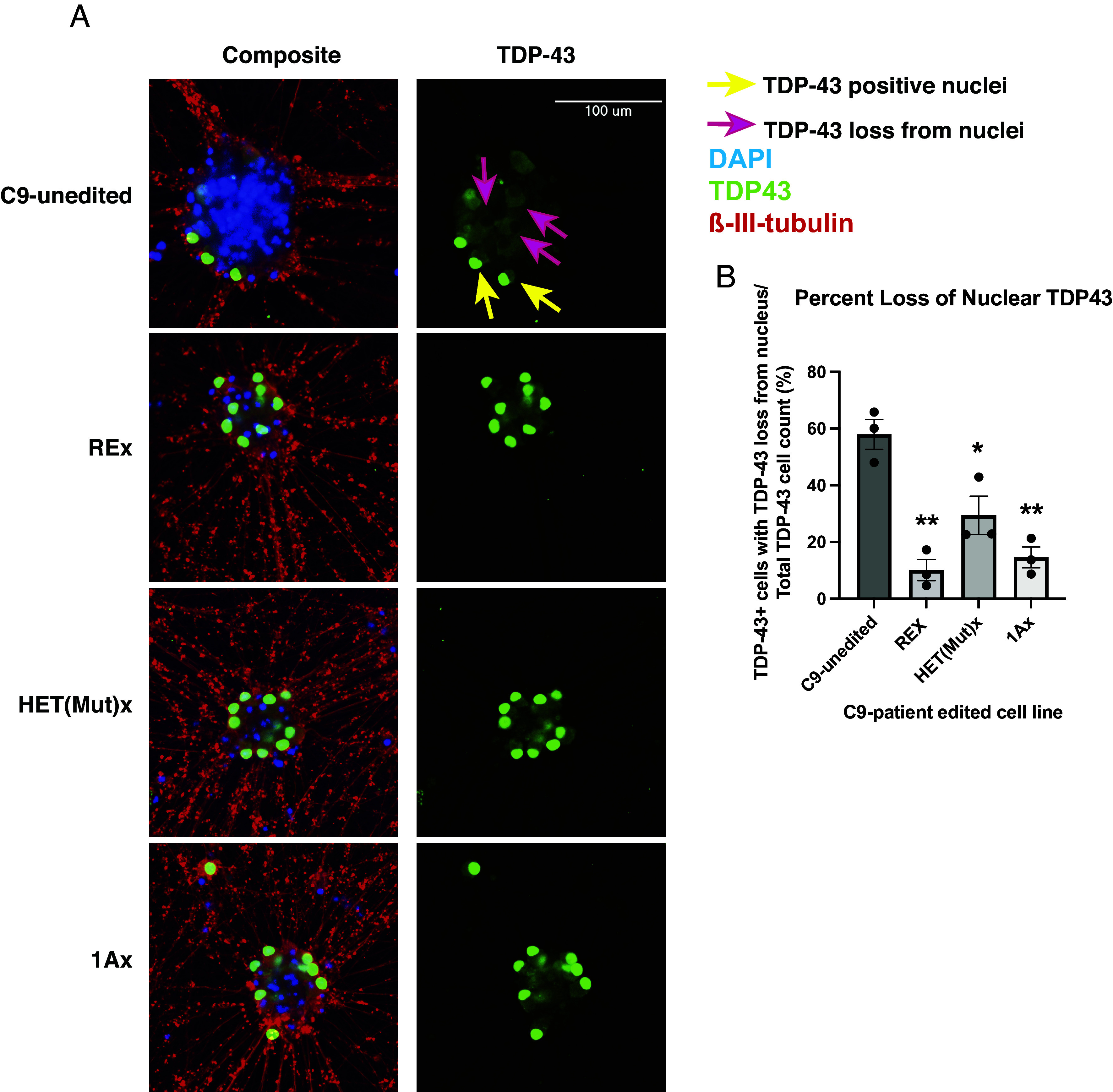
Three editing approaches ameliorate loss of nuclear TDP-43 in 7-wk-old neurons derived from C9 iPSC line. (*A*) Immunofluorescent images of neurons derived from unedited and edited C9 iPSCs. The neurons were grown for 7 wk and stained for TDP-43 (green), DAPI (blue) and beta-III-tubulin (red). Yellow arrow points to an example nuclei harboring TDP-43 and pink arrows to example TDP-43-positive cells whose nucleus are devoid of TDP-43. (*B*) Percentage of TDP-43-positive cells that lack nuclear TDP-43 (1-way ANOVA F(3,8) = 18.65; *P* < 0.001; *P < 0.05, **P < 0.01 by Tukey’s multiple comparison’s post hoc test between C9-unedited and each sample. REx, HET(Mut)x, and 1Ax were not statistically significantly different from one another). Each experiment contained three biologic replicates (separate wells). Error bars = SEM.

The pathological hallmark of C9-FTD/ALS is loss of nuclear TDP-43 and aggregation of cytoplasmic TDP-43 in affected neurons (43). These events are thought to be independent (43) and have been difficult to model in cellular and animal systems. Here we detected a clear loss of nuclear TDP-43 in 57% of TDP-43-positive motor neurons derived from our unedited patient iPSC line (Fig. 4*A* and *SI Appendix*, Fig. S13 pink arrows). This effect was age dependent, rising significantly 6 wk after induction of differentiation. The reason we could detect it is that we were able to maintain our motor neurons as a monoculture for 2 mo, after improving published protocols (29) for the generation of doxycycline-induced motor neurons by adding three growth factors (brain-derived neurotrophic factor (BDNF), glial cell line-derived neurotrophic factor (GDNF) and neurotrophin-3 (NT-3)). We quantified the loss of nuclear TDP-43 in the edited C9 lines that eliminated all RNA and DPR pathology (REx and HET(Mut)x) or silenced sense expression of the repeat expansion (1Ax) that at 7 wk postinduction. We found that on average, less than 30% of the TDP-43 positive cells lacked nuclear TDP-43 after removal of the repeat expansion (REx), the mutant allele (HET(Mut)x) or exon 1A (1Ax) (Fig. 4*B*). As the loss of nuclear TDP-43 was apparent only after aging motor neurons in culture for 7 wk postinduction, our system appears to recapitulate the interaction between age and genotype that is an important aspect of human C9-FTD/ALS. To our knowledge, TDP-43 pathology has not been detected in cultured human *C9orf72* mutant cells so far and has only been detectable in two other cell culture systems [GRN mutant (44) and TDP-43 (45) mutant cell lines] without artificially altering the TDP-43 expression and in the absence of drug treatment.

We noted that REx and HET(Mut)x cultures still harbored some motor neurons with nuclear loss of TDP-43, even though they lack the repeat expansion, and that removing ameliorated nuclear TDP-43 loss, even though it maintains antisense expression of the expanded repeat. Although a hallmark of C9-FTD/ALS, TDP-43 pathology is not specific to this gene mutation. It is also a classic pathological finding for other genetic and sporadic forms of FTD/ALS. In our cell culture system, we hypothesize the nuclear loss of TDP-43 is driven by the stress of aging to 7 wk, and exacerbated by the expression of the mutation. The take-away message is that the C9-unedited motor neurons had a greater pathological response to aging (i.e., more TDP-43-positive cells lost their nuclear TDP-43 expression) than did the edited cell motor neurons.

### Biallelic and Allele-Specific Excisions Improve Electrophysiological Function in 2- and 6-wk-old Motor Neurons.

We next measured the electrophysiologic effect of removing the repeat expansion in motor neurons. We examined population-level electrophysiology using multielectrode arrays (MEAs) at 3 wk postinduction and single neuron function using whole-cell patch-clamp at 6 wk postinduction. The difference in timepoints used for these analyses was necessitated by differing rates of electrophysiological maturation in iPSC-derived neuronal cultures maintained at different culture densities; high densities for MEA recordings and low, subconfluent densities for whole-cell patch-clamp recordings.

On MEAs, burst firing is defined as a period of activity in which the time between detected depolarizing spikes (interspike interval) is less than 100 ms for a minimum of ten consecutive spikes. Increased burst activity is an indicator of increasing functional maturity in cultured neurons (46–48). Furthermore, network bursts indicate detection of simultaneous burst activity across multiple electrodes in a single array and suggest the formation of synaptic networks in cultured neurons.

**Fig. 5.**
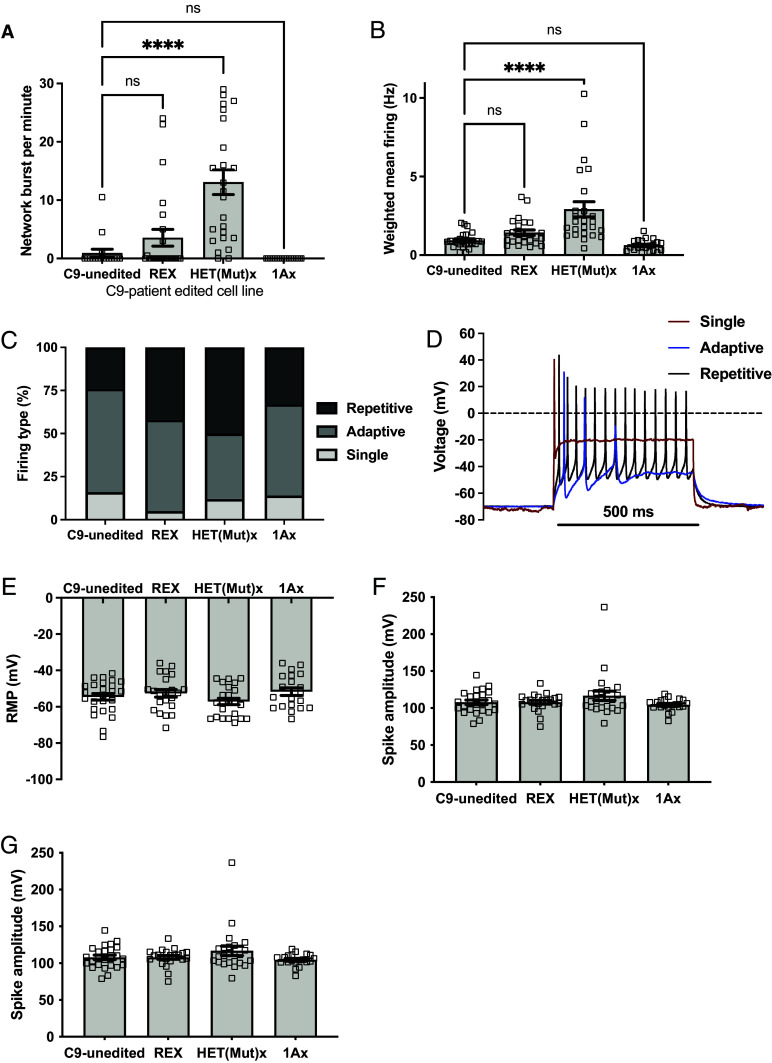
Improved in vitro neuronal electrophysiological function after removal of the *C9orf72* mutation. (*A*) C9-unedited and 1Ax 3-wk-old induced neurons showed minimal to no network bursting activity on MEAs (defined as burst events detected simultaneously from multiple electrodes within a single array). REx and HET(Mut)x showed network burst activity, with HET(Mut)x activity significantly increased compared to C9-unedited (1-way ANOVA F(3,84) = 17.3, *P* = 0.0003; ****P < 0.0001 by Tukey’s multiple comparison’s post hoc test). (*B*) Mean spontaneous firing rates weighted for electrodes showing activity on MEAs was significantly increased in HET(Mut)x compared to C9-unedited (1-way ANOVA F(3,89) = 13.3; *P* < 0.0001; ****P < 0.0001 by Tukey’s multiple comparison’s post hoc test). (*C* and *D*) Whole-cell patch clamp was used to identify the activity of individual neurons 40 d postinduction. Firing activity was measured during a 500 ms, 2 nA depolarizing current injection. Cells were binned into those firing a single action potential (single; *D*, red line), more than 1 action potential but with cessation of activity before 500 ms (adaptive; *D*, blue line), or repetitive firing that lasted the entire 500 ms (repetitive, *D*, black line). (*C*) Repetitive firing was increased, and adaptive firing decreased, in REx and HET(Mut)x compared to C9-unedited (χ^2^ test = 21.1 (6 degrees of freedom); *P* < 0.01). (*E*–*G*) There were no statistical differences between groups for individual action potential metrics, including (*E*) resting membrane potential (1-way ANOVA F(3,86) = 1.53; *P* = 0.2), (*F*) spike amplitudes (1-way ANOVA F(3,86) = 1.71; *P* = 0.17) and (*G*) action potential durations at 90% repolarization (APD_90_) (Kruskal–Wallis = 6.12; *P* = 0.11). Error bars = SEM.

The C9-unedited and 1Ax motor neurons showed no network burst activity at 3 wk of age; in contrast, both REx and HET(Mut)x motor neurons did demonstrate network burst activity (Fig. 5*A*), indicating that removing the repeat expansion, but not just silencing sense expression, enhances functional maturity and network forming capabilities in cultured motor neurons. Despite a lack of network activity, C9-unedited and 1Ax motor neurons were still spontaneously active (Fig. 5*B*). Spontaneous mean firing rate activity was nearly doubled (1.4 ± 0.17 Hz) in REx motor neurons and tripled (2.92 ± 0.48 Hz) in HET(Mut)x motor neurons compared to C9-unedited motor neurons (0.93 ± 0.11 Hz) (Fig. 5*B*).

It has been previously shown that *C9orf72* mutant iPSC-derived motor neurons have a decreased capacity to fire repetitive action potential trains compared with controls (49). Such data indicate that poor firing capacity is a phenotypic identifier for C9-ALS neurons in culture and suggest that interventions that increase firing capacity may therefore have therapeutic benefit. Using whole-cell patch clamp, we found that 500 ms, 2 nA current injections led to an increase in the percentage of induced motor neurons capable of repetitive firing among motor neurons in which the mutant repeat had been removed compared to C9-unedited [Fig. 5*C*, dark gray, REx, HET(Mut)x)] but not among motor neurons in which sense transcription is silenced (Fig. 5*C*, 1Ax). This increase in repetitive firing capacity occurred at the single-cell level and was not dependent on network activity. Nor was it dependent on individual action potential characteristics, as the motor neurons in each group showed similar resting membrane potentials (Fig. 5*E*), spike amplitudes (Fig. 5*F*), and action potential durations (Fig. 5*G*).

We did not compare the C9-isogenic series to the WT-series on electrophysiology (or TDP-43 pathology) because the genetic backgrounds between the two series would be confounding. We have shown previously that baseline electrophysiology differs significantly between iPSC-derived motor neurons with different genotypes, even in the absence of disease-causing mutations (48). These differences necessitate electrophysiological comparisons be performed between isogenic groups. Previous work has shown that iPSC-derived motor neurons harboring *C9orf72* and *TDP-43* mutations exhibit reductions in repetitive firing behavior by patch (49) and burst firing on MEAs (45). Therefore, we interpret the capacity for targeted edits that remove the repeat expansion to improve these metrics in our C9-mutant line as an improvement in overall functional performance.

In summary, we found an improvement of in vitro electrophysiologic markers of neuronal functional capacity for network activity and repetitive firing after removal of the repeat expansion biallelically (REx) or in an allele-specific manner (HET(Mut)x). This effect highlights that deficits due to the *C9orf72* mutation affect adaptive neuronal function, as the C9-unedited motor neurons demonstrated normal single-spike action potential properties at the single-neuron level.

## Discussion

Expression of the *C9orf72* locus is complex, with multiple transcription start sites and RNA splice forms, and translation from both the sense and antisense strands. Our eleven edited lines across two genetic backgrounds reveal important insights into the regulation of *C9orf72*. We demonstrate that transcription of 1A-transcripts is upregulated at least 10-fold on the mutant relative to the WT allele, using our quantitative allele-specific ddPCR methods. An upregulation of exon 1A-containing transcripts has previously been shown in postmortem human brain tissue (36), and our findings further this insight by demonstrating that upregulation is occurring from the mutant, but not WT, allele. Thus, transcriptional upregulation of the mutation is a possible biological driver of disease. We estimate that 30% or more of the total sense transcript from the *C9orf72* locus contains the mutation (based on the gap in detectable RNA in Fig. 1*C*, C9-unedited). To this, we must add antisense mutant transcripts, which may also contain the repeat expansion. In all, our observations suggest that induced motor neurons harboring the mutation produce a high amount of mutant RNA.

While RNAs harboring large repeat expansions could be toxic in themselves, as well as via their associated DPRs, our experiments suggest that at least a portion of these transcripts splice out the repeat expansion. How efficient this splicing event is, and what becomes of the spliced-out repeats is unknown at this point. However, boosting this innate splicing event (14, 50, 51) could constitute a viable therapeutic approach.

Our work shows that C9orf72 is haplo-sufficient in motor neurons, since motor neurons containing a single copy of the gene produce the same amount of protein as do WT motor neurons. Our data on haplo-sufficiency of *C9orf72* at the protein level in motor neurons is significant because a dominant view in the field is that *C9orf72* is haplo-insufficient (18, 37, 52, 53). However, this possibility is not supported by population genetic (loss-of-function mutations exist in the human population but are not known to present with FTD/ALS) or by heterozygous and homozygous knock-out animal models (23, 24, 26, 54–56), which lack neurologic phenotypes. Now we have cellular confirmation of haplo-sufficiency in motor neurons. Nevertheless, artificially reducing *C9orf72* function can exacerbate toxicity from the mutant allele (18, 21, 25), suggesting a protective role of WT *C9orf72* gene expression. It is possible that downregulation of the *C9orf72* RNA or protein in neurons or other cell types, or accelerated metabolism of either, occur in the context of age or disease state and exacerbate the toxic gain-of-function drivers of disease. This may explain why RNA (1, 36, 57, 58) and protein (53, 59–61) levels have been found to be low in patient tissues. But as a field we should be cautious in interpreting these observations as evidence that C9orf72 disease results from haplo-insufficiency, since the cumulative genetic, animal model and now human motor neuron cellular data instead support haplo-sufficiency.

Part of the confusion regarding haplo-sufficiency of *C9orf72* may also be attributable to quantification of RNA rather than protein changes. We show that reduction in RNA by over half after removal of one allele (HET(Mut)x or HET(WT)x, Fig. 1 *G* and *H*) does not alter protein levels (Fig. 2 *A*–*D*) in motor neurons. A second potential explanation is that quantification of C9orf72 protein has been flawed because of nonspecific antibodies. We (28, 39) and others (62) have shown, using knock-out lines, that many of the antibodies commonly used in the field are not specific for *C9orf72* despite showing signal in various assays. Given the nonspecificity of many antibodies for C9orf72 protein, it may be time to reexamine protein expression levels in postmortem and other human tissues.

Another major insight of our work is that sense transcription and the expression of sense RNA-derived poly-GA is allele-independent but the production of poly-GP dipeptide off the mutant allele is influenced by the WT allele. Approximately one-third of poly-GP protein is from the antisense strand, based on poly-GP production in the 1Ax motor neurons (Fig. 3*C*), in which there is no sense transcription (Fig. 1 *C*, *E*, and *G*). Removal of the WT allele increases the amount of poly-GP further, but without altering poly-GA levels (Fig. 3*B*). If loss of the WT allele altered the stability of dipeptide repeats globally, we would expect both poly-GA and poly-GP levels to rise in the HET(WT)x. Furthermore, the difference in poly-GP amounts between 1Ax and HET(WT)x is not due to a change in measurable antisense RNA levels, as antisense RNA levels were comparable between these two lines (Fig. 3*D*). We conclude that the upregulation of poly-GP reflects other cellular factors, such as preferential stabilization of the poly-GP peptides, increased translation of antisense transcripts or the presence of additional antisense transcripts not measured by our assay. Regardless of the underlying mechanisms, this surprising finding provides a potential explanation for the failure of ASOs to reverse pathology. ASOs target sense transcription, and are thought to suppress sense RNAs from both the WT and mutant alleles given their sequence homology to both alleles (15, 63, 64). In turn, suppression of sense RNAs by ASOs could facilitate the translation of antisense RNAs. Our observation merits further investigation but encourages caution when developing new therapeutics.

We show that TDP43 pathology can be revealed in iPSC-derived motor neurons carrying the *C9orf72* mutation when they are aged; up to now, TDP43 was not detectable without directly manipulating the TDP43 pathway, and many thought it was not possible to see TDP43 pathology in cell culture. While we demonstrate an interaction between mutation and cellular age on the appearance of TDP-43 pathology, the other ingredient may be cellular stress induced by prolonged time in culture. We propose that our culture system may provide a means to investigate why patients who are born with the mutation and express it throughout the brain and body only develop the disease later in life.

An interesting observation is that while neither REx nor HET(Mut)x motor neurons have sense or antisense expression of the repeat expansion (which was removed in both lines), and both produce the same amount of C9orf72 protein, they differ in their ability to ability to rescue the TDP-43 (Fig. 4*B*) and electrophysiologic (Fig. 5 *A*–*C*) phenotypes. Biallelic excision of the intronic region containing the repeat expansion (REx) had a larger effect on TDP-43 pathology than did the 21 kb excision of the mutant allele in HET(Mut)x, which, by contrast, increased spontaneous and repetitive firing to a larger degree. We wonder whether the 21 kb excision removed regulatory regions on the mutant allele that normally limit firing capacity of the motor neurons. Our results suggest there is more to be learned about the influence of the intronic and regulatory regions of the *C9orf72* gene in nondiseased and diseased states.

Our experimental design avoided a number of confounding factors that could have muddled the interpretation of our findings. One is the effect of genetic background, which we minimized by comparing three edits head-to-head in isogenic cell lines, all derived from the same patient’s iPSCs. The power of isogenic series to isolate the effects of specific genomic changes has been demonstrated particularly effectively by the iNDI project (65) to compare neurodegenerative disease–causing mutations on a single cell line. A limitation of this study is that our biological insight result primarily from a series of edits made in a single patient cell line. While our experiments will need to be replicated with cells from different patients and from lines with varying repeat lengths, isogenic comparisons are the current gold standard for head-to-head comparisons of genetic changes. Another confounding factor is the possibility that diseased cells influence the health of edited cells, for instance by secreting pathogenic factors such as DPRs. For this reason, we think another gold standard should be to compare genomic changes between clonal cell lines, as we have done here, rather than between unsorted cultures.

Our results have important therapeutic implications. Up to now, the *C9orf72* locus has been challenging to edit in a manner compatible with clinical translation. Others have shown that CRISPR editing of the *C9orf72* locus reduced pathologic burden in cells and mice (66, 67), but both approaches disrupted the normal allele in addition to the mutant allele (by biallelic excision of either exon 1A or exon 1B). This may be deleterious as homozygous knockout causes early lethality in mice (23–25) and as we have shown here that excision of exon 1A silences sense transcription but does not eliminate all pathology. In particular, it did not eliminate the production of toxic peptides from antisense transcripts and did not improve electrophysiologic measures. In contrast, our two other approaches (biallelic excision of the repeat expansion, or selective excision of the mutant allele) preserved WT protein levels, corrected RNA abnormalities, abrogated dipeptide repeat production, reduced TDP43 pathology, and improved adaptive electrophysiologic function in patient-derived iPSC-derived motor neurons. From these data, we can advance repeat expansion excision and allele-specific excision to further preclinical testing, including in postmitotic neurons and in vivo models.

## Methods

### Cell Line Generation and Editing.

We used a deidentified patient iPSC line harboring the *C9orf72* mutation and a control cell line without mutation (WT-control). We first knocked in the inducible motor neuron transcription factor transgene cassette in the CLYBL safe-harbor locus. We then edited the lines using HiFi spCas9 (Macrolabs, UC Berkeley) and two gRNAs (*SI Appendix*, Table S1). We used PacBio single-molecule sequencing to size the repeat expansion, detect repeat expansion excision in C9-REx and detect methylation after editing across C9-patient lines. Further details on iPSC cell line generation (*SI Appendix*, Figs. S1–S11), maintenance, single-molecule sequencing, and motor neuron differentiation are detailed in *SI Appendix, Supplemental Methods*.

### RNA Quantification by ddPCR.

500 ng of RNA from 2-wk-old induced motor neurons was run on the QX100 Droplet Reader (Bio-Rad 186-3002) with three technical replicates of each of three biologic replicates (independent wells of differentiated motor neurons) using primers and probes in *SI Appendix*, Table S2. See *SI Appendix, Supplemental Methods* for full details.

### C9orf72 Protein Quantification by WES.

C9orf72 protein quantification by WES (Bio-Techne) was performed according to the manufacturer’s protocol and primary antibodies mouse anti-C9orf72 (GeneTex, GTX634482 at 1:100 and rabbit anti-GAPDH (AbCam, AB9485) at 1:1,000. Duplexed secondaries included 9.5 µL of mouse (ProteinSimple, DM-002) and 0.5 µL of 20× anti-rabbit (ProteinSimple, 043-426). See *SI Appendix, Supplemental Methods* for full details.

### Dipeptide Repeat Quantification by MDS ELISA Immunoassay.

We used the Small Spot Streptavidin Plate (L45SA, MSD). Poly-GA was detected using anti-GA antibody (MABN889, Millipore) at 1 µg/mL (capture) and 2 µg/mL (detect) final concentration and 18 µg total protein per sample (blocking buffer A, solution PBS). Poly-GP was detected using anti-GP antibody (affinity purified TALS828.179 from TargetALS, purification lot A-I 0757 and stock concentration 1.39 mg/mL) at a final concentration of 2 µg/mL capture and 4 µg/mL detected with 18.5 µg total protein per sample (blocking buffer A, solution TBS). See *SI Appendix, Supplemental Methods* for full details.

### TDP-43 Immunocytochemistry and Quantification.

TDP-43 immunocytochemistry and quantification was performed in fixed 7-wk-old induced motor neurons. Primary antibodies: rabbit anti-TDP43 (10782-2-AP, Proteintech) at 1:500, beta-III-tubulin (480011, Invitrogen) at 1:250 incubated overnight at 4 °C. Secondary antibodies: goat anti-rabbit Alexa Fluor 488 nm and Goat anti-mouse Alexa Fluor 594 nm incubated at room temperature for 1 h. DAPI (D1306, ThermoFisher Scientific) for 5 min at room temperature. See *SI Appendix, Supplemental Methods* for full details.

### Electrophysiology.

Detailed protocols for induced motor neuron MEA and whole-cell patch clamp are available in *SI Appendix, Supplemental Methods*.

## Supplementary Material

Appendix 01 (PDF)

## Data Availability

Sequencing data have been deposited in NIH Bioproject; GEO (PRJNA1058535; GSE252200) (35, 38). All study data are included in the article and/or *SI Appendix*.
