# Supplementary material for: Reversal of C9orf72 mutation-induced transcriptional dysregulation and pathology in cultured human neurons by allele-specific excision
Source: Proc Natl Acad Sci U S A. 2024 Apr 15;121(17):e2307814121. doi: 10.1073/pnas.2307814121 (PMC11047104; doi:10.1073/pnas.2307814121)
Supplement: Supplementary file 1 — Appendix 01 (PDF) [file pnas.2307814121.sapp.pdf]

PNAS

Supporting Information for

Reversal of *C9orf72* mutation-induced transcriptional dysregulation and pathology in cultured human neurons by allele-specific excision

Sachdev A[1], Gill K[1,2], Sckaff M[1,2], Birk AM[1], Aladesuyi Arogundade O[2,3], Brown KA[2,3], Chouhan RS[2,3], Issagholian-Lewin PO[2,3], Patel E[2,3], Watry HL[1], Bernardi MT[1], Keough KC[1], Tsai Y-C[4], Smith AST[5,6], Conklin BR[#1,6], Clelland CD[#2,3].

[1]Gladstone Institutes, San Francisco, California, USA
[2]Weill Institute for Neurosciences, University of California San Francisco, San Francisco, California, USA
[3]Memory & Aging Center, Department of Neurology, University of California San Francisco, San Francisco, California, USA
[4]Pacific Biosciences, Menlo Park, California, USA
[5]Department of Physiology and Biophysics, University of Washington, Seattle, Washington, USA
[6]The Institute for Stem Cell and Regenerative Medicine, University of Washington, Seattle, Washington, USA
[7]Departments of Medicine, Ophthalmology, and Pharmacology, University of California San Francisco, San Francisco, California, USA.

#Correspondance: Claire D. Clelland: claire.clelland@ucsf.edu

This PDF file includes:

Methods
References for methods
Tables S1 to S2
Figs. S1 to S13

Methods

Cell line generation, maintenance and determination of editing efficiencies. We used a de-identified patient iPSC line(1) harboring the *C9orf72* mutation and a control cell line without mutation(2) (WT-control). We maintained iPSCs in mTesR plus plated on Matrigel (Corning 356231), passaging at 60-80% confluency. All cell lines had a normal karyotype and negative quarterly mycloplasma testing.

We first knocked-in the inducible motor neuron transcription factor transgene cassette(3, 4) in the CLYBL safe-harbor locus of a C9-patient line using spCas9 and ATGTTGGAAGGATGAGGAAA gRNA. This transgene includes human NGN2, ISL1, LHX3 (hNIL) under the TET operator and is inducible by doxycycline, mCherry (for positive selection) and neomycin antibiotic resistance (for negative fluorescence). Red-fluorescing cells were sorted via FACS to isolate single, live cells. Each resulting clonal cell line was analyzed for incorporation of the transgene in the CLYBL locus by PCR (left homology arm junction primers CAGACAAGTCAGTAGGGCCA and AGAAGACTTCCTCTGCCCTC) with preservation of one of the alleles (CLYBL wild-type primers TGACTAAACACTGTGCCCCA and AGGCAGGATGAATTGGTGGA). We used Copy Number Variation (CNV) ddPCR to pick a clone with a single transgene insertion of the hNIL plasmid (Nemomycin primers CATGGCTGATGCAATGCG and TCGCTTGGTGGTCGAATG, probe FAM; Primers UBE2D2 – Bio-Rad 10031255, probe HEX) to mitigate the risk of integration of the transgene at genomic loci other than CLYBL. Expression of neuronal markers was confirmed 2-weeks after doxycycline induced differenation in parental C9-unedited and WT-unedited lines by RT-PCR for ChAT (forward TGAGTACTGGCTGAATGACATG and reverse AGTACACCAGAGATGAGGCT primers) and HB9 (forward GCACCAGTTCAAGCTCAAC and reverse GCTGCGTTTCCATTTCATCC primers).

To engineer each iPSC line we used HiFi spCas9 protein (Macolabs, UC Berkeley) and two gRNAs (**Table S1**) to create an excision, using our published protocol(5). gRNAs were designed to have no exact off-target matches and the lowest predicted off-targets using CRISPOR (Homo sapiens – USCS Dec. 2013 (GRCh38/hg38)). gRNAs were ordered from IDT or Synthego. Cas9-gRNA RNP (spCas9 (40μM), sgRNA (100μM)) was delivered by nucleofection (Lonza AAF-1002B, Lonza AAF-1002X, Pulse Code = DS138) to 350,000 iPSCs suspended in 20 μl of P3 Buffer. The cells were recovered with mTesR plus supplemented with ROCK1 inhibitor (Selleckchem S1049) at 10 μM and Clone R (Stemcell 05888). Approximately 50% of iPSCs died within the first 24 hours of electroporation, as expected. Following a 48-72 hour recovery, we collected the pool of edited cells and either hand-picked 48 clones or sorted single live cells via FACS to a single well on a 96-well plate. Single cell sorting was performed using a BD FACSAria Fusion (Beckton Dickinson) by the Gladstone Flow Cytometry Core. The QC alignment of each laser was verified with Cytometer Setup and Tracking Beads (Becton Dickinson) before sample acquisition. A forward scatter threshold of 15,000 was set to eliminate debris from list mode data, and a fixed number of events was collected. In some experiments mCherry fluorescence (excitation 561nm, emission 610nm) was also used to define sorting parameters. Drop delay determination and 96-well plate set-up setup was done using Accudrop beads (Becton Dickinson). Gating on forward scatter area versus height and side scatter area versus height was used to make the single cell determination. The specifications of the sort layout included single cell precision, 96-well collection device and target event of 1. After cultures reached 60-70% confluency, each well

was split into two wells of a new 48- or 96-well plate, one for sequencing and the other to continue the cell line. We screened clones based on the presence of an excision band using PCR (primers and expected band size from **Table S1**). We also performed PCR across each the 5' and 3' cut site (**Table S1**), with one primer site located inside the excision region, to ensure absence of a band (for homozygous edits) or presence of the WT allele (for heterozygous edits). For all lines except C9-REx, we then Sanger sequenced the excision band (MCLAB, Quintara). If the sequence was ambiguous (i.e., had overlapping nucleotide reads at the same mapped nucleotide position) we subcloned the line to achieve clone purity and clean sequencing. All lines were karyotyped (WiCell or Cell Line Genetics) after editing. We additionally compared Sanger sequencing across potential predicted off-target sites in each of our therapeutically edited clonal lines with the unedited parental line after PCR amplification using primers in **Fig. S11**. For C9-REx we could not use this approach since PCR could not amplify the large repeat expansion, and hence could not distinguish clones with excision of both the mutant and WT allele from clones with excision of the WT allele only. Therefore, we used single-molecule sequencing of clonal REx lines to determine the percentage of clones with an excision of the repeat expansion region (as described below).

PacBio single molecule sequencing to size the repeat expansion, detect repeat expansion excision (C9-REx) and detect methylation. Because polymerase amplification fails to accurately size the entirety of the *C9orf72* GC rich repetitive region, we use single molecule sequencing(6) of a genomic region containing the repeat region. We collected high molecular weight DNA using Genomic Tip (Qiagen 10243) and confirmed absence of smearing by running the DNA on a 1.5% agarose gel. The Gladstone Genomics Core performed library preparation according to the "No Amp Targeted Sequencing" protocol using 3-5 μg of DNA per sample as measured by Qubit. Briefly, we blocked the free ends of purified genomic DNA and then excised the gene region of interest using spCas9, a gRNA targeting 5' to the repeat expansion (GGAAGAAAGAATTGCAATTA) and a gRNA targeting 3' to the repeat expansion (TTGGTATTTAGAAAGGTGGT). Excising the genomic region harboring the repeat expansion yields a 3639 bp fragment from the WT allele and a fragment of variable size from the mutant allele depending on the size of the GGGGCC repeat. We then ligated adapters and barcodes to blunt free ends and sequenced 3-5 barcoded lines per SMRT Cell on either a Sequel I or Sequel II sequencer. We used a 3-pass filter such that each molecule of DNA had to be sequenced at least 3 times to be included in analysis. HiFi reads were generated using the CCS analysis included in SMRTLink v13. The CCS SMRT analysis was executed with options to include 5mC basecalls and to process all reads. PacBio's pb-CpG-tools v2.3.2 was used with "--pileup-mode count" to generate combined proportions of predicted 5mC methylation.

iPSC differentiation into motor neurons. We used the hNIL transgene cassette TET-on system in the CLYBL safe-harbor locus of a C9-patient line and WT-control line. Introduction of doxycycline for 3 days induced the expression of 3 human transcription factors: NGN2, ISL1, LHX3. We followed the previously published protocol(3, 4) with notable exceptions, including higher concentrations of the growth factors BDNF, GDNF and NT-3 (each at 20 ng/ml). Our detailed protocol is published(7).

RNA quantification by ddPCR. 2-week-old induced motor neurons were lysed with papain (Worthington LK003178) and RNA was isolated using Quick-RNA Microprep Kit (Zymo R1051). cDNA was synthesized using iScript™ Reverse Transcription Supermix (Biorad 1708841) and

500 ng of RNA. ddPCR was run with 3 technical replicates of each of 3 biologic replicates (independent wells of differentiated motor neurons) on the QX100 Droplet Reader (Bio-Rad 186-3002)(8). Each ddPCR reaction consisted of 12.5 uL of 2x SuperMix for Probes (no dUTP) (Bio-Rad 186-3024), primer/probe (see **Table S2**), 5 ng of cDNA, and nuclease-free water up to 25 µL. Droplets were generated with QX 100 Droplet Generator (Bio-Rad 186-3001) and 20 µL of the reaction mixture with 70 µL of oil. The ddPCR reactions were run in a Deep Well C1000 Thermal Cycler (Bio-Rad 1851197) with the following cycling protocol: (1) 95°C for 10 min; (2) 94°C for 30 s; (3) 58°C for 1 min; (4) steps 2; and 3 repeat 39 times; (5) 98°C for 10 min; (6) hold at 4°C. We thresholded positive samples as those with >10 positive droplets to avoid error due to noise. We quantified positive droplets for each target and normalized the amount to our loading control (*UBE2D2*) (Bio-Rad QuantaSoft™ Analysis Pro Software). We chose this housekeeping gene because its expression level remained stable across iPSCs and differentiated neurons.

For allele-specific expression of exon 1A- and 1B-containing transcripts, we utilizing a coding SNP in the exon 2 splice acceptor (rs10757668) in our patient line. We centered our ddPCR probe on this SNP and used the same primers as above to amplify the exon 1A-exon 2 and exon 1B-exon 2 junctions (**Table S2**). Expression from each allele was quantified in a single reaction and reported as a ratio.

C9orf72 protein quantification by Simple Western. We performed protein quantification by streptavidin-based Simple Western capillary reaction (WES; Bio-Techne) according the manufacturers protocol (Jess & Wes Separation Module SM1001 to SM1012), with the following specifications: protein was collected from cultured motor neurons 2 weeks post-induction in RIPA buffer with protease inhibitor and sonicated for 5 min, and denatured at 90°C for 10 min. 0.3 µg/µl protein from each sample was mixed with 1 µl 5x Master Mix and 0.1x Sample Buffer (EZ Standard Pack PS-ST01EZ-8) to a total volume of 5 µl. 3 µl of this mix was loaded per sample onto a 12-230 kDa plate (ProteinSimple SM-W004-1). Primary antibodies were mouse anti-C9orf72 (GeneTex, GTX634482) at a 1:100 dilution and rabbit anti-GAPDH (AbCam, AB9485) at 1:1000 dilution (total volume 10 µl per lane). Duplexed secondaries included 9.5 µl of mouse (ProteinSimple, DM-002) and 0.5 µl of 20x anti-rabbit (ProteinSimple, 043-426) per lane. Reaction times: 25 min separation time at 375 V, 5 min antibody dilutant time, 30 min primary antibody, 30 min secondary antibody; quantification at 4 seconds of detection (high dynamic range). Under these optimized conditions, each antibody produced a single peak at 57 kDa (C9orf72) and 42 kDa (GAPDH). Area under the curve was quantified for each peak and C9orf72 AUC was normalized to GAPDH AUC for each sample. Averages across 3 biological replicates (independent wells of neuronal differentiation) of motor neurons from each edited cell line aged 14 days post-induction were compared to the average protein expression of their respective unedited controls.

Dipeptide repeat quantification by Meso Scale Discovery (MDS) ELISA immunoassay. We followed the manufacturer's protocol for the Small Spot Streptavidin Plate (L45SA, MSD). Poly-GA was detected using anti-GA antibody (MABN889, Millipore) at 1 µg/ml (capture) and 2 µg/ml (detect) final concentration and 18 µg total protein per sample (blocking buffer A, solution PBS). Poly-GP was detected using anti-GP antibody (affinity purified TALS828.179 from TargetALS, purification lot A-I 0757 and stock concentration 1.39 mg/ml). TALS828.179 (A-I 0757) anti-GP antibody was used at a final concentration of 2 µg/ml capture and 4 µg/ml detect with 18.5 µg total

protein per sample (blocking buffer A, solution TBS). The plate was coated with capture antibody overnight at 4°C with no agitation. The plate was blocked with 3% MSD Blocker A (R93BA, MSD) in 1X DPBS for 1 hour at 750 rpm, then incubated for 1 hour with protein lysate at 750 rpm at room temperature. Detection antibody was added after the lysate for 1 hour. Washes were performed between steps thrice with 1X DPBS + 0.05% Tween-20. MSD Read Buffer A (R92TG, MSD) was added to the plate before being immediately placed in the MSD Model 1250 Sector Imager 2400 plate reader. Signal was calculated by comparing luminescence intensity for each control or edited patient line to background (*i.e.,* C9-KO line), data was presented as a fold change above C9-KO baseline/background level. Our detailed MSD ELISA immunoassay protocol is published(9).

TDP-43 immunocytochemistry and quantification. Seven-week-old motor neurons were fixed by adding 4% PFA directly to culture media for 30 min followed by 3 PBS washes of 10 min each. Cells were permeabilized by 1X DPBS 0.1% Triton-X in 3 washes of 10 min each at room temperature and blocked with 1X DPBS 0.1% Triton-X + 5% BSA for 1 hour at room temperature. Primary antibodies: rabbit anti-TDP43 (10782-2-AP, Proteintech) at 1:500, beta-III-tubulin (480011, Invitrogen) at 1:250. Primary antibodies were incubated overnight at 4°C. Secondary antibodies included Goat anti-rabbit Alexa Fluor 488 nm and Goat anti-mouse Alexa Fluor 594 nm. Secondary antibodies were incubated at room temperature for 1 hour. DAPI (D1306, ThermoFisher Scientific) was added to the penultimate of five, 5 min PBS washes. Our detailed immunocytochemistry protocol is published(10). After staining, motor neurons were scanned on the ImageXpress Micro Confocal (Molecular Devices). TDP-43 expressing motor neurons were quantified using Elements AI (Imaging Software NIS-Elements AR 5.30.04 64-bit). We trained the software to differentiate TDP-43-positive cells with or without nuclear TDP-43 on images from independent differentiations not included in the quantification. A blinded observer hand-classified and hand-counted TDP-43-positive cells and confirmed the trends detected by AI. We counted all TDP-43-positive cells with loss of nuclear TDP-43 and divided this number by the number of TDP-43-positive cells to arrive at the percentages shown in Fig. 4B.

Electrophysiology. Motor neurons cultured for whole-cell patch clamp analysis were mounted on the 37°C heated stage of an inverted DIC microscope (Nikon) connected to an EPC10 patch clamp amplifier and computer running Patchmaster software (HEKA). Cultures were bathed in a Tyrode's solution containing 140 mM NaCl, 5.4 mM KCl, 1.8 mM CaCl2, 1 mM MgCl2, 10 mM glucose, and 10 mM HEPES (all from Sigma-Aldrich). An intracellular recording solution containing 120 mM L-aspartic acid, 20 mM KCl, 5 mM NaCl, 1 mM MgCl2, 3 mM Mg2+-ATP, 5 mM EGTA, and 10 mM HEPES (all from Sigma-Aldrich) was loaded into borosilicate glass patch pipettes (World Precision Instruments) with a resistance in the range of 3–6 MΩ. Before a gigaΩ seal was formed, all offset potentials were nulled. In all recordings, the fast and slow capacitance was compensated. Membrane potentials were corrected by subtraction of a 30 mV tip potential, calculated using the HEKA software. Motor neurons that required more than 100 pA of current to achieve a −70 mV resting membrane potential were excluded as excessive application of current is indicative of poor patch quality and/or membrane integrity. Likewise, any recordings that failed to maintain a seal resistance of at least 400 megaΩ were excluded from downstream analysis.

Single action potentials were generated via application of a 5 ms, 2 nA current pulse. Depolarization-evoked repetitive firing was achieved via application of a series of 500 ms current injections starting at −30 pA and increasing in 10 pA increments. Both single action potentials and repetitive firing behavior were recorded in current-clamp mode. Gap-free recordings of spontaneous activity in patched motor neurons were performed in current-clamp mode for 30 s with 0 pA current injection to provide a measure of the resting membrane potential held by the motor neuron without current input. All recordings and analyses of action potential waveforms

Population level function in iPSC-derived motor neuron cultures was assessed in 48-well MEA plates using the Maestro MEA system (Axion Biosystems). Motor neurons were maintained at 37°C/5% CO_2 throughout the 1-minute recording period and the Maestro system's standard settings were employed for all recordings. Spike detection thresholds were set at 5× the standard deviation of the noise and network burst detection was recorded if at least 25% of the electrodes in a given well showed synchronous activity. Reported results were calculated by averaging all of the electrodes in each well, then averaging data from duplicate wells. In total, 14 to 16 wells (collected across 3 independent differentiation runs) were analyzed for each experimental group.

Data availability

Single molecule sequencing data are available at GEO GSE252200 and BioProject PRJNA1058535.

References for methods (from supplementary information)

1. M. Pribadi, *et al.*, CRISPR-Cas9 targeted deletion of the C9orf72 repeat expansion mutation corrects cellular phenotypes in patient-derived iPS cells. *BioRxiv* (2016) https:/doi.org/10.1101/051193.

2. Y. Miyaoka, *et al.*, Isolation of single-base genome-edited human iPS cells without antibiotic selection. *Nat. Methods* **11**, 291–293 (2014).

3. M. S. Fernandopulle, *et al.*, Transcription Factor-Mediated Differentiation of Human iPSCs into Neurons. *Curr. Protoc. Cell Biol.* **79**, e51 (2018).

4. C. M. Feliciano, *et al.*, Allele-Specific Gene Editing Rescues Pathology in a Human Model of Charcot-Marie-Tooth Disease Type 2E. *Front. Cell Dev. Biol.* **9**, 723023 (2021).

5. K. Gill, A. Sachdev, B. Conklin, C. D. Clelland, Creating iPSC lines with Ribonucleoprotein (RNP): Nucleofection, Single-cell Sorting, Genotyping, and Line Maintenance Protocol. *Protocols.io*, dx.doi.org/10.17504/protocols.io.4r3l2oeopv1y/v1 (2022).

6. M. T. W. Ebbert, *et al.*, Long-read sequencing across the C9orf72 “GGGGCC” repeat expansion: implications for clinical use and genetic discovery efforts in human disease. *Mol. Neurodegener.* **13**, 46 (2018).

7. M. Sckaff, *et al.*, Differentiation of iPSCs with the hNIL construct into motor neurons protocol v1. Protocols.io, dx.doi.org/10.17504/protocols.io.14egn76kqv5d/v1 (2022).

8. K. Jing Kay Lam, O. Arogundade, C. D Clelland, Copy number variation analysis by ddPCR v1. Protocols.io, 10.17504/protocols.io.q26g7pkd8gwz/v1 (2024).

9. K. Gill, M. Sckaff, C. D. Clelland, MSD gold streptavidin antibody preparation and plate run protocol v1. Protocols.io, dx.doi.org/10.17504/protocols.io.5qpvobkj9l4o/v1 (2022).

10. M. Sckaff, C., *et al.*, Immunocytochemistry of motor neurons derived from iPSCs with the hNIL construct protocol v1. Protocol.io, 10.17504/protocols.io.6qpvr68bovmk/v1 (2022).

			spCas9 gRNA		Excision Primers			5' Cut Site Primers			3' Cut Site Primers		
**Edit Name**	**Edit**	**Excision Size (WT allele)**	**5' gRNA**	**3' gRNA**	**F primer**	**R primer**	**Expected excision band amplicon length (bp)**	**F primer**	**R primer**	**Expected 5' cut site amplicon length (bp)**	**F primer**	**R primer**	**Expected 3' cut site amplicon length (bp)**
**REx**	repeat expansion excision	7bp if no RE, 25 bp if 3 repeats	AACTCAGGAGTCGCGCGCTA	ggcccgccccgaccacgccc	CCGCTAGGAAAGAGAGGTGCG	GAGGAGAGCCCCCGCTTCTAC	505 (unedited WT allele), unedited RE will not amplify, 480 (edited)	N/A	N/A	N/A	N/A	N/A	N/A
**1Ax**	exon 1A excision	227bp	TGCGATGACGTTTTCTCACG	TACTGTGAGAGCAAGTAGTG	GATCCAGCAGCCTCCCCTAT	GCTACAGGCTGCGGTTGTTT	440 (unedited), 214 (edited)	TCCAGCAGCCTCCCCTATT	TTTACGTGGGCGGAACTTGT	216	AGAGAGGTGCGTCAAACAGC	CTCCTGAGTTCCAGAGCTTGC	282
**1Bx**	exon 1B excision	124bo	CGTGGTCGGGGCGGGCCCGG	GCTGTTTGGGGTTCGGCTGC	TACTCGCTGAGGGTGAACAAG	CAGTCGCTAGAGGCGAAAGC	390 (unedited), 266 (edited)	N/A	N/A	N/A	GTGGCTGTTTGGGGTTCGG	CCAGTCGCTAGAGGCGAAAG	137
**HET(Ref)x**	Allele-specific excision of REFERENCE allele	21,044bp	CTCTGTGAGAAGTTTTTATC	GACTTAGAAGAAATATTGTG	AGGAACCAAGCAGCCATGAA	GGGAAGCCACACCCTTGTAA	Unedited will not amplify (21436), 392 (edited)	CTTTGGCACAGATAGGCCAC	GGCAGGGTGACTGCTTTAAC	199	TGCCCAGAATAAATTTTGGATAACT	GGGAAGCCACACCCTTGTAA	337
**HET(Alt)x**	Allele-specific excision of ALTERNATE allele	21,044bp	CTCTGTGAGAAGTTCTTATC	GACTTAAAAGAAATATTGTG	AGGAACCAAGCAGCCATGAA	GGGAAGCCACACCCTTGTAA	Unedited will not amplify (21436), 392 (edited)	CTTTGGCACAGATAGGCCAC	GGCAGGGTGACTGCTTTAAC	199	TGCCCAGAATAAATTTTGGATAACT	GGGAAGCCACACCCTTGTAA	337
**KO (WT line)**	Excision from 5' to exon 1A through exon 2	7,205bp	TGTGCGAACCTTAATAGGGG	AATGGGGATCGCAGCACATA	GCAGACCAAAAGACGCAAGG	ACCAGAAAATAAGCTTTCAACAGAT	Unedited will not amplify (7748), 578 (edited)	GCAGACCAAAAGACGCAAGG	CAGCGAGTACTGTGAGAGCA	751	GGGTTAGGGGCCAAATCTCC	ACCAGAAAATAAGCTTTCAACAGAT	932
**KO (patient line)**	Excision from 5' to exon 1A to exon 3-4 intron (using all 4 allele-specific gRNAs in 1 reaction)	21kb	CTCTGTGAGAAGTTTTTATC and CTCTGTGAGAAGTTCTTATC	GACTTAGAAGAAATATTGTG and GACTTAAAAGAAATATTGTG	AGGAACCAAGCAGCCATGAA	GGGAAGCCACACCCTTGTAA	Unedited will not amplify (21436), 392 (edited)	CTTTGGCACAGATAGGCCAC	GGCAGGGTGACTGCTTTAAC	199	TGCCCAGAATAAATTTTGGATAACT	GGGAAGCCACACCCTTGTAA	337

Supplementary Table 1. **Guide RNAs and primers used to generate and verify, respectively, each edited cell line.** We used spCas9 with a protospacer adjacent motif (PAM) of NGG (not included in the gRNA sequence). We ran PCR products on a gel using the primers listed for each line to verify the emergence of an excision band (excision primers) at the expected band size or to sequence across the cut sites (5' or 3' cut site primers). For homozygous lines we expect the presence of an excision band and the absence of both 5' and 3' cut site bands. For heterozygous lines we expect an excision band on the edited allele and preservation of the WT cut site bands on the unedited allele. Clean sequencing for each PCR product further confirms the absence of indels and clone purity. Outcomes of each of these PCRs and sequencing reactions are depicted for each line in SF1-SF10. Excision size using the listed gRNAs is provided for each type of excision in the WT line (excisions of the repeat expansion can vary depending on repeat expansion length). Expected amplicon size for each set of PCR primers is also provided.

ddPCR Design	Probe target	Catalog Name (Cat #) Vendor	Primer sequence or MIQE	Probe sequence	Fluorophore
1A-long transcript (variant 3)	Exon 1A-exon 2 junction (NM_001256054.2)	Hs00948764_m1 for C9orf72 Thermo (4351372)	MIQE: GGTGTCCCGCTAGGAAAGAGAGGTGCGTCAAACAGCGAC AAGTTCCGCCCACGTAAAAGATGACGCTTGGTGTGTCAG CCGTCCCTGCTGCCCGGTTGCTTCTCTTTTGGGGGCGGG GTCTAGCAAGAGCAGGTGTGGGTTTAGGAGATATCTCCG GAGCATTTGGATAATGTGACAGTTGGAATGCAGTGATGTC GACTCTTTGCCCACCGCCATCTCCAGCTGTTGCCAAGAC AGAGATTGCTTTAAGTGGCAAATCACCTTTATTAGCAGCT ACTTTTGCTTACTGGGACAATATTCT (amplicon length 102)	GTTTAGGAGATAT CTCCGGAGCATT	FAM
1B transcript (variant 2)	Exon 1B-exon 2 junction (NM_018325.4)	ID: AII1NCR, Name:C9_NEWV2 Thermo (4331348)	F primer: CGGTGGCGAGTGGATATCTC<br>R primer: TGGGCAAAGAGTCGACATCA	TAATGTGACAGTT GGAATGC	FAM
Exon 2-3 (total sense mRNA)	Exon 2-3 junction (total mRNA)	Hs00376619_m1 Thermo (4448892)	MIQE: TTAATCTTTGATGGAAACTGGAATGGGGATCGCAGCACAT ATGGACTATCAATTATACTTCCACAGACAGAACTTAGTTTC TACCTCCCACTTCATAGAGTGTGTGTTGATAGATTAACAC ATATAATCCGGAAAGGAAGAATATGGATGCATAAGGAAAG ACAAGAAAATGTCCAGAAGATTATCTTAGAAGGCACAGAG AGAATGGAAGATCAGGGTCAGAGTATTATTCCAATGCTTA CTGGAGAAGTGATTCCTGTAATGGAACTGCTTTCATCTAT GAAATCACACAGTGTTCCTG<br>(amplicon length 123)	AGAATATGGATGC ATAAGGAAAGAC	VIC
1A Allele Specific	Exon 1A-exon 2 junction (NM_001256054.2) targeting SNP rs10757668 in exon 2 splice acceptor	AN7DZHR Thermo (4332077)	F primer: GCAGGTGTGGGTTTAGGAGATATC<br>R primer: ACATCACTGCATTCCAACTGTCA	CGGAGCATTTGGA TAAT (mutant allele) CGGAGCATTTAGA TAAT (WT allele)	FAM/VIC
1B Allele Specific	Exon 1B-exon 2 junction (NM_018325.4) targeting SNP rs10757668 in exon 2 splice acceptor	AN9HU3N Thermo (4332077)	F primer: CGGTGGCGAGTGGATATCTC<br>R primer: ACATCACTGCATTCCAACTGTCA	CGGAGCATTTGGA TAAT (mutant allele) CGGAGCATTTAGA TAAT (WT allele)	FAM/VIC
UBE2D2 HEX	Housekeeping gene (NM_003339, NM_181838)	UBE2D2, Human (HEX); dHsaCPE5036421 Biorad (10031255)	ATCACAGTGGTCTCCAGCACTAACTATTTCAAAAGTACTC TTGTCCATCTGTTCTCTGTTGTGTGATCCCAATCCAGATG ATCCTTTAGTGCCTGAGATTGCTCGGATCTACAAAACAGA TAG<br>(amplicon length 65)	Included in amplicon (MIQE)	HEX
UBE2D2 FAM	Housekeeping gene NM_003339, NM_181838	UBE2D2, Human (FAM) dHsaCPE5036420 Biorad (10031252)	ATCACAGTGGTCTCCAGCACTAACTATTTCAAAAGTACTC TTGTCCATCTGTTCTCTGTTGTGTGATCCCAATCCAGATG ATCCTTTAGTGCCTGAGATTGCTCGGATCTACAAAACAGA TAG<br>(amplicon length 65)	Included in amplicon (MIQE)	FAM
AS	Antisense	IDT (304211718)	F primer: GGTTCGCTAGGAACCCGAGACGGTC<br>R primer: GCATTGCTGCCCTCATATGC	CCGACACCCAGCT TCGGTCAGAG	FAM

Supplementary Table 2. Exon-spanning ddPCR probes used in Figures 1 and 3.

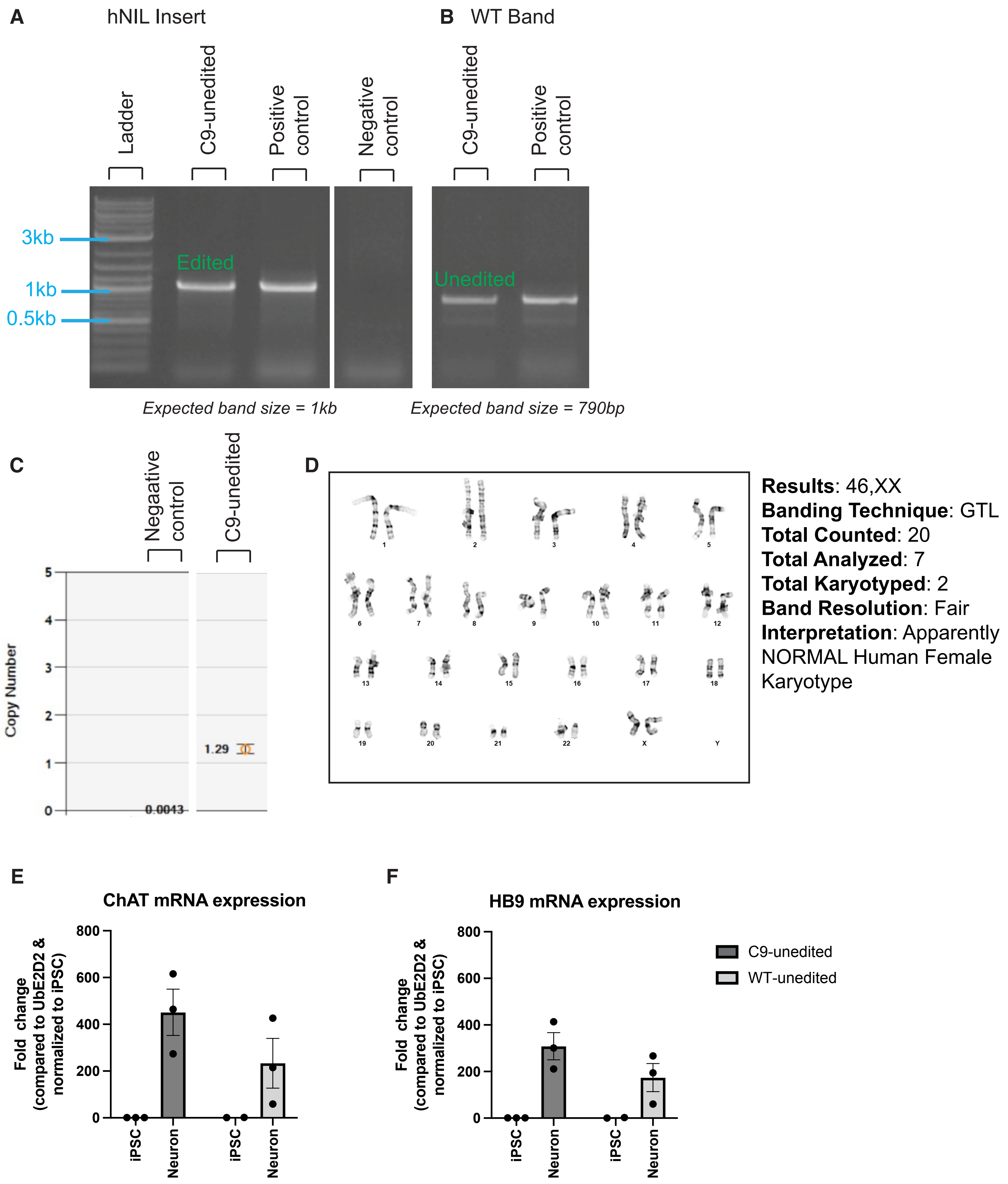
A
hNIL Insert
Ladder
C9-unedited
Positive control
Negative control
3kb
1kb
0.5kb
Edited
Expected band size = 1kb
B
WT Band
C9-unedited
Positive control
Unedited
Expected band size = 790bp
C
Negaative control
C9-unedited
Copy Number
5
4
3
2
1
0
0.0043
1.29
D
Results: 46,XX
Banding Technique: GTL
Total Counted: 20
Total Analyzed: 7
Total Karyotyped: 2
Band Resolution: Fair
Interpretation: Apparently NORMAL Human Female Karyotype
E
ChAT mRNA expression
Fold change (compared to UbE2D2 & normalized to iPSC)
800
600
400
200
0
iPSC
Neuron
iPSC
Neuron
F
HB9 mRNA expression
Fold change (compared to UbE2D2 & normalized to iPSC)
800
600
400
200
0
iPSC
Neuron
iPSC
Neuron
C9-unedited
WT-unedited

Supplementary Figure 1. **Construction of a C9 unedited cell line with the hNIL cassette**. **(A)** Band corresponding to the insertion of the hNIL construct into an unedited patient cell line (called C9-unedited), as determined by PCR. **(B)** Preservation of a WT band indicates the line is heterozygous. **(C)** ddPCR copy number assay shows 1 insertion of the hNIL construct. **(D)** The cell line had a normal karyotype. **(E, F)** Neurons derived from doxycycline-induced expression of the hNIL transcription factors show an average of 340-fold and 240-fold increase in expression of neuronal markers ChAT and HB9 mRNA transcript, respectively, 2 weeks after induction by doxycycline, relative to iPSCs, as measured by RT-PCR in both the unedited WT and patient cell lines.

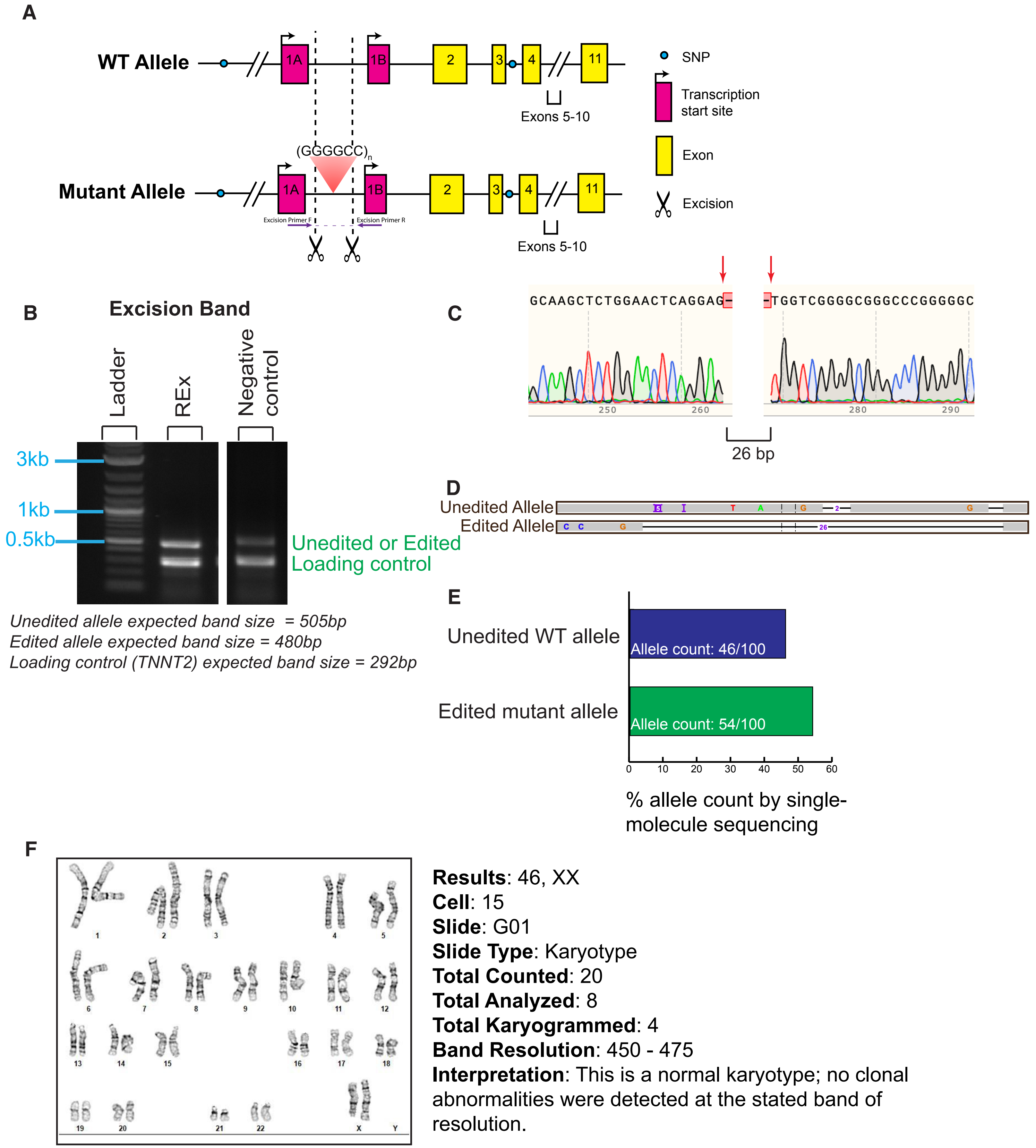
A
WT Allele
1A
1B
2
3
4
11
Exons 5-10
SNP
Transcription start site
Exon
Excision
(GGGGCC)n
Mutant Allele
Excision Primer F
Excision Primer R
B
Excision Band
Ladder
REx
Negative control
3kb
1kb
0.5kb
Unedited or Edited
Loading control
Unedited allele expected band size = 505bp
Edited allele expected band size = 480bp
Loading control (TNNT2) expected band size = 292bp
C
GCAAGCTCTGGAACTCAGGAG
TGGTCGGGGCGGGCCCGGGGGC
250
260
280
290
26 bp
D
Unedited Allele
Edited Allele
E
Unedited WT allele
Allele count: 46/100
Edited mutant allele
Allele count: 54/100
0
10
20
30
40
50
60
% allele count by single-molecule sequencing
F
Results: 46, XX
Cell: 15
Slide: G01
Slide Type: Karyotype
Total Counted: 20
Total Analyzed: 8
Total Karyogrammed: 4
Band Resolution: 450 - 475
Interpretation: This is a normal karyotype; no clonal abnormalities were detected at the stated band of resolution.

Supplementary Figure 2. Construction of the C9-REx cell line. **(A)** Position of the gRNAs (indicated by scissors) and excision primers (purple arrows) used to create and verify, respectively, excision of the repeat expansion (REx) in the *C9orf72* gene in a patient cell line. **(B-D)** The cell line had a band at ~500bp using excision primers (B) and clean Sanger sequencing (C; cut sites indicated by pink arrows). However, we could not tell from these data whether the line had a homozygous excision of the repeat region or a heterozygous excision of the WT allele only, since the expanded repeat region fails amplification and the unedited and edited band sizes are very similar in size. We therefore used single-molecule sequencing (D) to determine that the clone was pure, and carried a heterozygous excision of the expanded repeat leaving a 26 bp deletion on the mutant allele (using SNPs to differentiate alleles). **(E)** Allele count of PacBio sequencing data shows both alleles were equally covered by sequencing. **(F)** The cell line had a normal karyotype.

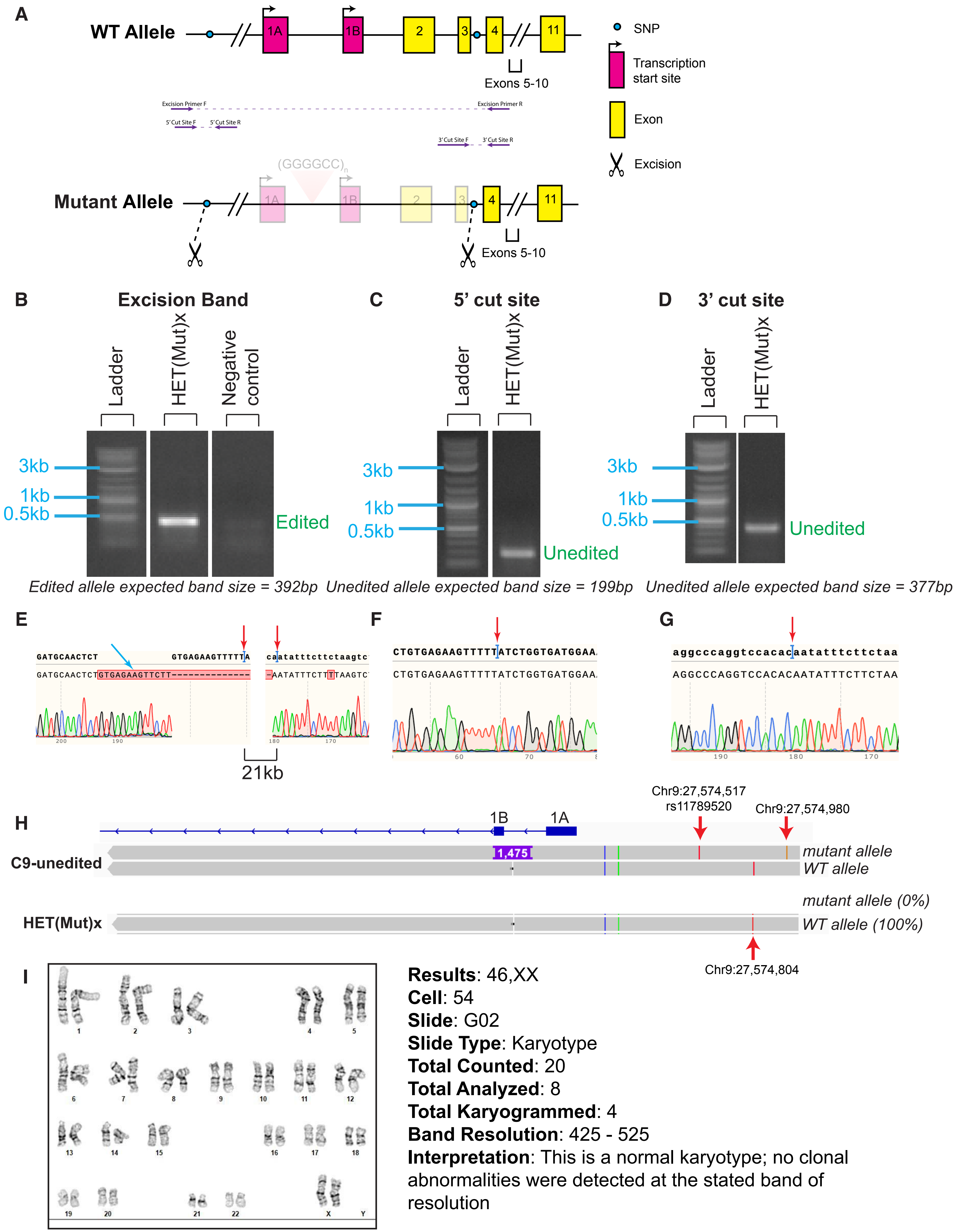

A
WT Allele
1A
1B
2
3
4
11
Exons 5-10
SNP
Transcription start site
Exon
Excision
Excision Primer F
Excision Primer R
5' Cut Site F
5' Cut Site R
3' Cut Site F
3' Cut Site R
(GGGGCC)n
Mutant Allele
B
Excision Band
Ladder
HET(Mut)x
Negative control
3kb
1kb
0.5kb
Edited
Edited allele expected band size = 392bp
C
5' cut site
Unedited
Unedited allele expected band size = 199bp
D
3' cut site
Unedited allele expected band size = 377bp
E
GATGCAACTCT GTGAGAAGTTTTTA caatatttcttctaagtc
21kb
F
CTGTGAGAAGTTTTTATCTGGTGATGGAA
G
aggcccaggtccacacaatatttcttctaa
AGGCCCAGGTCCACACAATATTTCTTCTAA
H
Chr9:27,574,517
rs11789520
Chr9:27,574,980
C9-unedited
1,475
mutant allele
WT allele
HET(Mut)x
mutant allele (0%)
WT allele (100%)
Chr9:27,574,804
I
Results: 46,XX
Cell: 54
Slide: G02
Slide Type: Karyotype
Total Counted: 20
Total Analyzed: 8
Total Karyogrammed: 4
Band Resolution: 425 - 525
Interpretation: This is a normal karyotype; no clonal abnormalities were detected at the stated band of resolution

Supplementary Figure 3. Construction of the C9-HET(Mut)x cell line. (A) Position of the gRNAs (indicated by scissors) and excision and cut site primers (purple arrows) used to create and verify, respectively, a ~22kb excision of the mutant *C9orf72* allele in a patient cell line. SNPs phased to the repeat expansion (blue dots) were used to target the mutant allele. **(B-D)** Presence of an excision band (B) and preservation of bands at both the 5' (C) and 3' (D) cut sites at the expected sizes indicate the line is a heterozygous excision. **(E-G)** Corresponding clean Sanger sequencing shows the clone is pure and there are no indels on the unedited (WT) allele (pink arrow – cut site; blue arrow – misaligned Sanger sequencing), which is further confirmed by (H). **(H)** Single-molecule PacBio sequencing in a 5kb region around the repeat expansion confirms heterozygosity of the HET(Mut)x cell line. Gene reads right to left, with exons 1A and 1B denoted. SNPs are used to distinguish alleles. Examples of the WT and mutant C9-unedited alleles are shown to illustrate how SNPs distinguish alleles. Blue (Chr9:27,574,016 Hg38; rs2131553112) and green (Chr9:27,574,089 Hg38; rs1373538) lines (SNPs) differ from reference but are homozygous between the two alleles and therefore not useful at differentiating alleles. The three red SNPs (2 on the mutant allele and 1 on the WT allele) differentiate between the two alleles. The repeat expansion is shown as a 1475 insertion (purple). The HET(Mut)x line has only one (WT) allele as denoted by the SNP pattern and has no indels around the repeat regions or the first two exons of the gene. **(I)** The cell line had a normal karyotype.

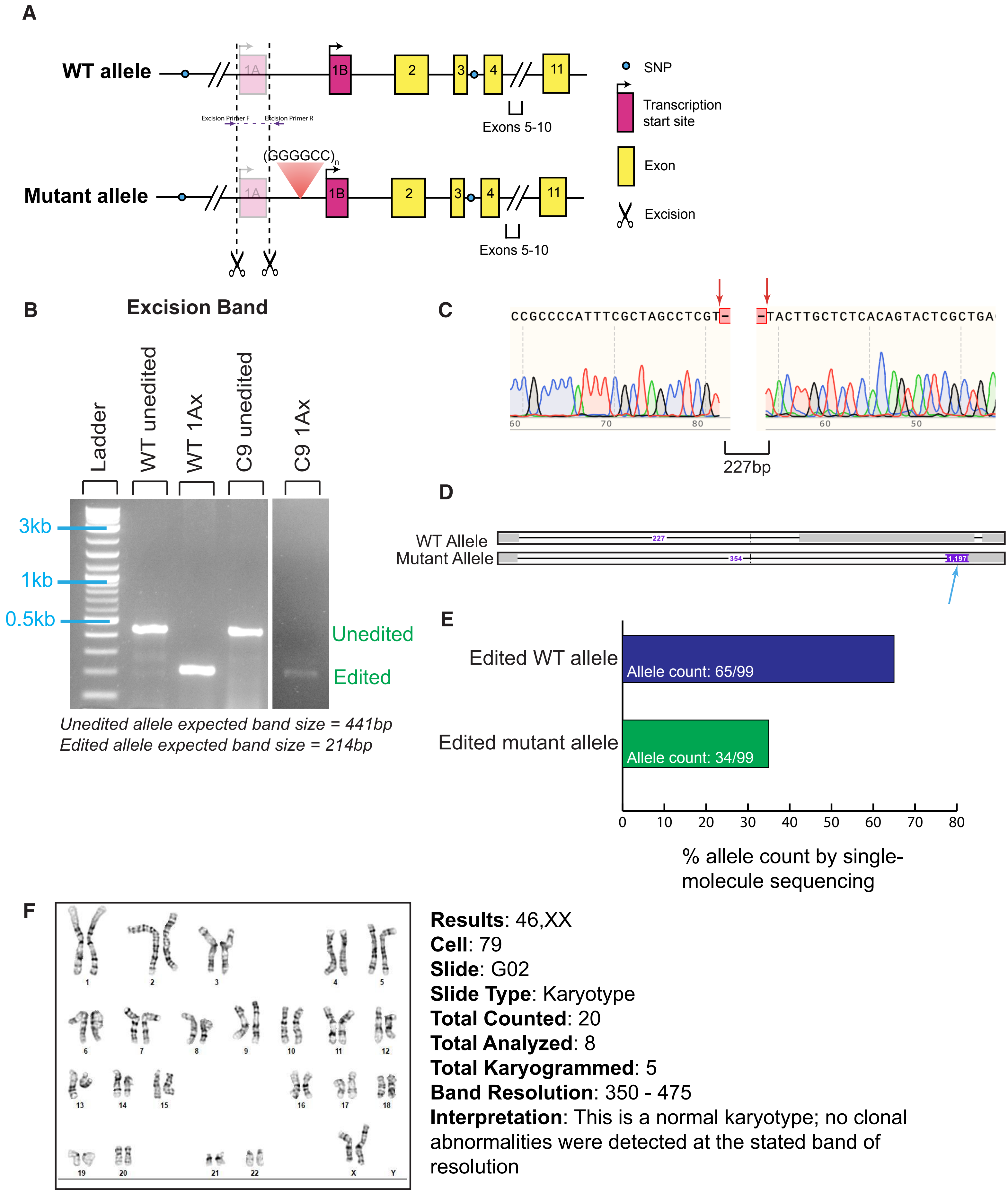
A
WT allele
1A
1B
2
3
4
11
Excision Primer F
Excision Primer R
Exons 5-10
(GGGGCC)n
Mutant allele
SNP
Transcription start site
Exon
Excision
B
Excision Band
Ladder
WT unedited
WT 1Ax
C9 unedited
C9 1Ax
3kb
1kb
0.5kb
Unedited
Edited
Unedited allele expected band size = 441bp
Edited allele expected band size = 214bp
C
CCGCCCCATTTCGCTAGCCTCGT-
-TACTTGCTCTCACAGTACTCGCTGA
227bp
D
WT Allele
Mutant Allele
227
354
1,197
E
Edited WT allele
Allele count: 65/99
Edited mutant allele
Allele count: 34/99
0 10 20 30 40 50 60 70 80
% allele count by single-molecule sequencing
F
Results: 46,XX
Cell: 79
Slide: G02
Slide Type: Karyotype
Total Counted: 20
Total Analyzed: 8
Total Karyogrammed: 5
Band Resolution: 350 - 475
Interpretation: This is a normal karyotype; no clonal abnormalities were detected at the stated band of resolution

Supplementary Figure 4. Construction of the C9-1Ax cell line. (A) Position of the gRNAs (indicated by scissors) and excision primers (purple arrows) used to create and verify, respectively, excision of exon 1A of the *C9orf72* gene in a patient cell line. **(B)** Presence of an excision band at the expected size and absence of a WT band in C9-1Ax indicates the line is homozygous. WT-unedited and C9-unedited serve as negative controls and WT-1Ax serves as a positive control. **(C)** Sanger sequencing shows the excision cut sites (pink arrows). **(D)** Single-molecule sequencing revealed 227 bp excision on the WT allele and a 354 bp excision on the mutant allele (blue arrow shows the repeat expansion on the mutant allele). **(E)** Total alleles sequenced by single molecule sequencing showed a modest preference for the WT allele, as expected. **(F)** The cell line had a normal karyotype.

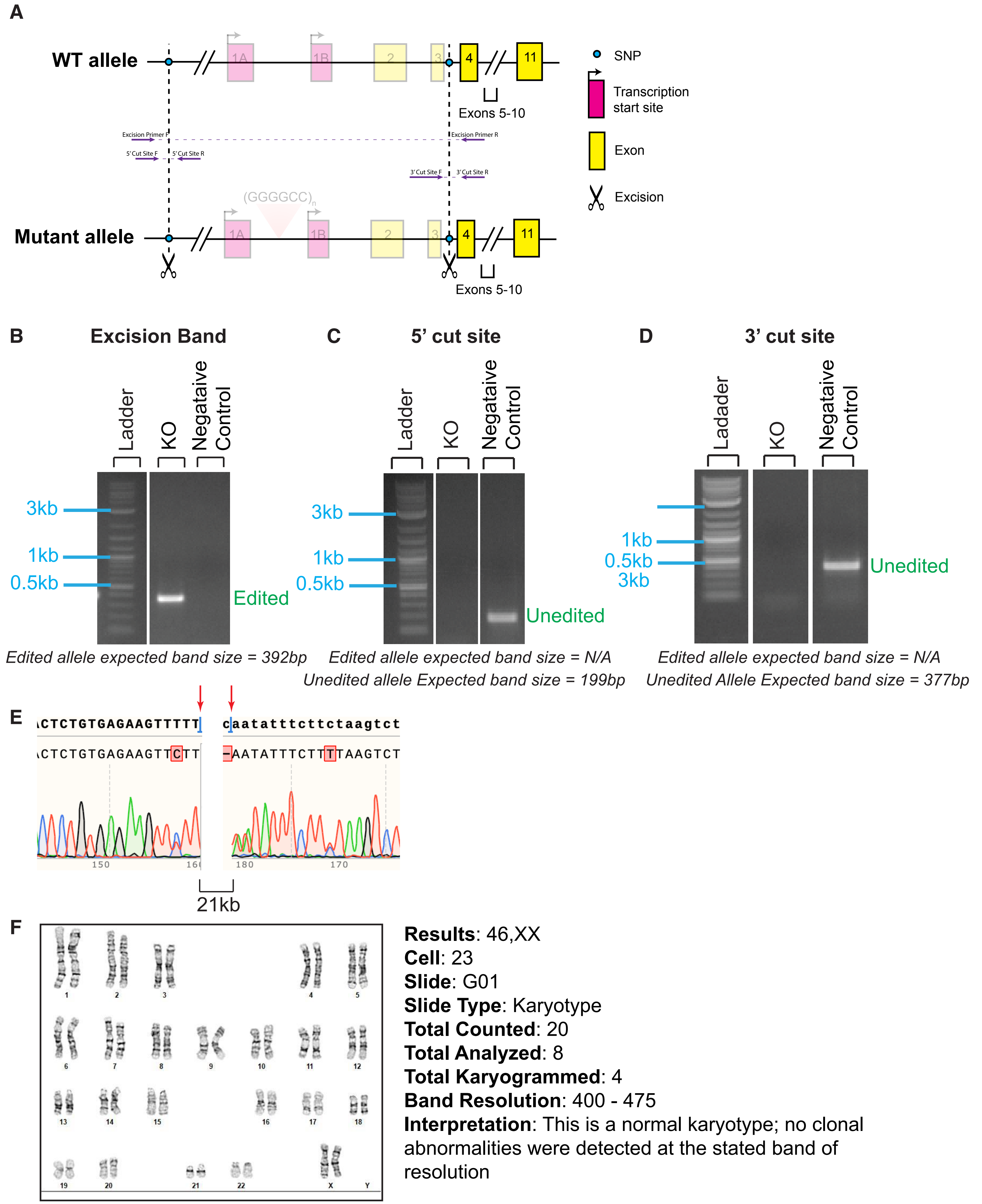
A
WT allele
1A
1B
2
3
4
11
Exons 5-10
Excision Primer F
Excision Primer R
5' Cut Site F
5' Cut Site R
3' Cut Site F
3' Cut Site R
(GGGGCC)n
Mutant allele
SNP
Transcription start site
Exon
Excision
B
Excision Band
Ladder
KO
Negataive Control
3kb
1kb
0.5kb
Edited
Edited allele expected band size = 392bp
C
5' cut site
Unedited
Edited allele expected band size = N/A
Unedited allele Expected band size = 199bp
D
3' cut site
Ladader
1kb
0.5kb
3kb
Edited allele expected band size = N/A
Unedited Allele Expected band size = 377bp
E
CTCTGTGAGAAGTTTTT
caatatttcttctaagtct
CTCTGTGAGAAGTTCTT
-AATATTTCTTTTAAGTCT
150
160
180
170
21kb
F
1
2
3
4
5
6
7
8
9
10
11
12
13
14
15
16
17
18
19
20
21
22
X
Y
Results: 46,XX
Cell: 23
Slide: G01
Slide Type: Karyotype
Total Counted: 20
Total Analyzed: 8
Total Karyogrammed: 4
Band Resolution: 400 - 475
Interpretation: This is a normal karyotype; no clonal abnormalities were detected at the stated band of resolution

Supplementary Figure 5. Construction of the C9-KO cell line. (A) Position of the gRNAs (indicated by scissors) and excision and cut site primers (purple arrows) used to create and verify, respectively, a ~21 kb excision of both the mutant and WT *C9orf72* alleles in a patient cell line. Line was made by using 4 allele-specific gRNAs targeting SNPs (blue circles) in one electroporation reaction. **(B-D)** Presence of an excision band at the expected size (B) and absence of WT cut site bands (C,D) indicate homozygosity. **(E)** Sanger sequencing shows the excision cut sites (pink arrows). **(F)** The cell line had a normal karyotype.

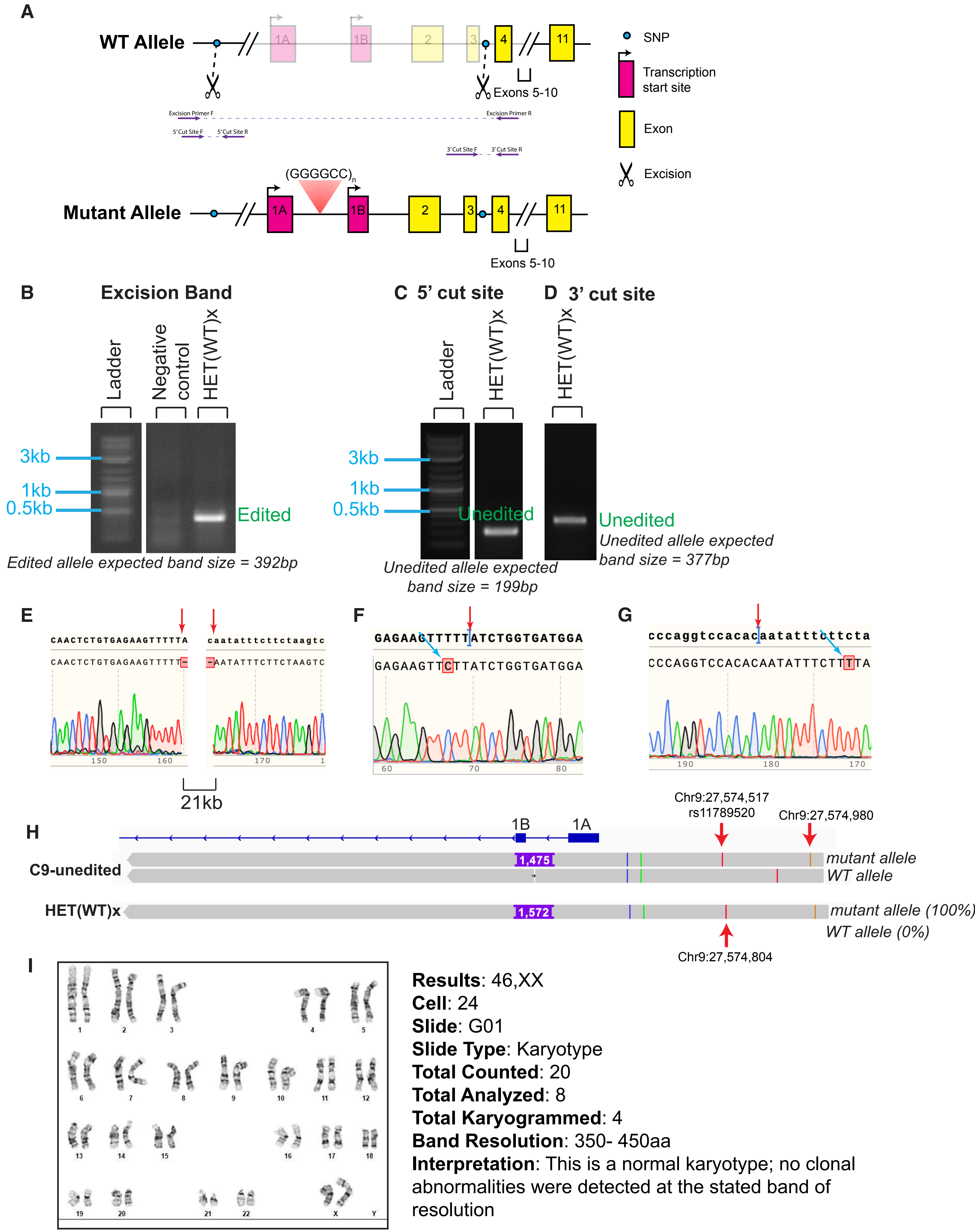
A
WT Allele
1A
1B
2
3
4
11
Exons 5-10
Excision Primer F
Excision Primer R
5' Cut Site F
5' Cut Site R
3' Cut Site F
3' Cut Site R
(GGGGCC)n
Mutant Allele
SNP
Transcription start site
Exon
Excision
B
Excision Band
Ladder
Negative control
HET(WT)x
3kb
1kb
0.5kb
Edited
Edited allele expected band size = 392bp
C
5' cut site
Unedited
Unedited allele expected band size = 199bp
D
3' cut site
Unedited allele expected band size = 377bp
E
CAACTCTGTGAGAAGTTTTTA
caatatttcttctaagtc
CAACTCTGTGAGAAGTTTTT-
-AATATTTCTTCTAAGTC
21kb
F
GAGAAGTTTTTATCTGGTGATGGA
GAGAAGTTCTTATCTGGTGATGGA
G
cccaggtccacacaatatttcttcta
CCCAGGTCCACACAATATTTCTTTTA
H
Chr9:27,574,517
rs11789520
Chr9:27,574,980
C9-unedited
1,475
mutant allele
WT allele
HET(WT)x
1,572
mutant allele (100%)
WT allele (0%)
Chr9:27,574,804
I
Results: 46,XX
Cell: 24
Slide: G01
Slide Type: Karyotype
Total Counted: 20
Total Analyzed: 8
Total Karyogrammed: 4
Band Resolution: 350- 450aa
Interpretation: This is a normal karyotype; no clonal abnormalities were detected at the stated band of resolution

Supplementary Figure 6. Construction of the C9-HET(WT)x cell line. (A) Position of the gRNAs (indicated by scissors) and excision and cut site primers (purple arrows) used to create and verify, respectively, excision of the WT *C9orf72* allele in the C9-patient cell line. The SNPs phased to the WT allele (blue dots) were targeted to create the 21 kb excision. **(B-D)** Presence of an excision band at the expected size (B) and preservation of bands at both the 5' (C) and 3' (D) cut sites at the expected sizes indicate the line has a heterozygous excision. **(D-G)** Corresponding clean Sanger sequencing shows the clone is pure and there are no indels on the unedited (mutant) allele (pink arrow – cut site; blue arrow – SNP), which is further confirmed by (H). **(H)** Single-molecule PacBio sequencing in a 5kb region around the repeat expansion confirms heterozygosity of the HET(WT)x cell line. Gene reads right to left, with exons 1A and 1B denoted. SNPs are used to distinguish alleles. Examples of the WT and mutant C9-unedited alleles are shown to illustrate how SNPs distinguish alleles. Blue (Chr9:27,574,016 Hg38; rs2131553112) and green (Chr9:27,574,089 Hg38; rs1373538) lines (SNPs) differ from reference but are homozygous between the two alleles and therefore not useful at differentiating alleles. The three red SNPs (2 on the mutant allele and 1 on the WT allele) differentiate between the two alleles. The repeat expansion is shown as an insertion (purple). The HET(WT)x line has only one (mutant) allele as denoted by the SNP pattern and has no indels around the repeat expansion (purple) or the first two exons of the gene. **(I)** The cell line had a normal karyotype.

A

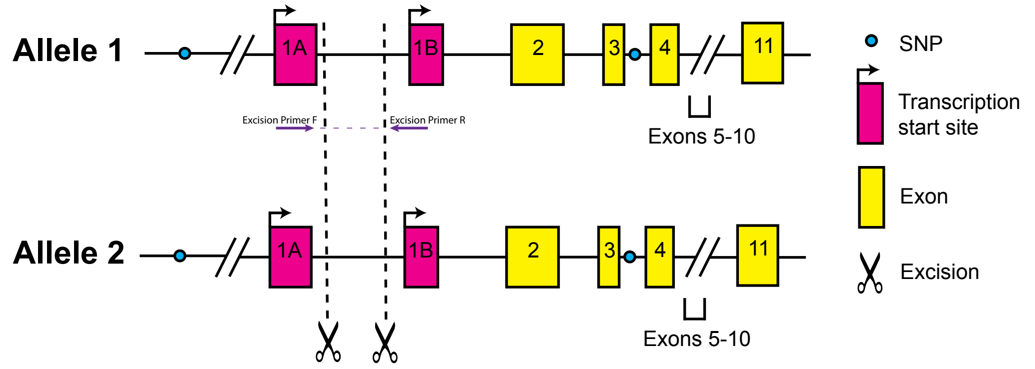

B Excision Band

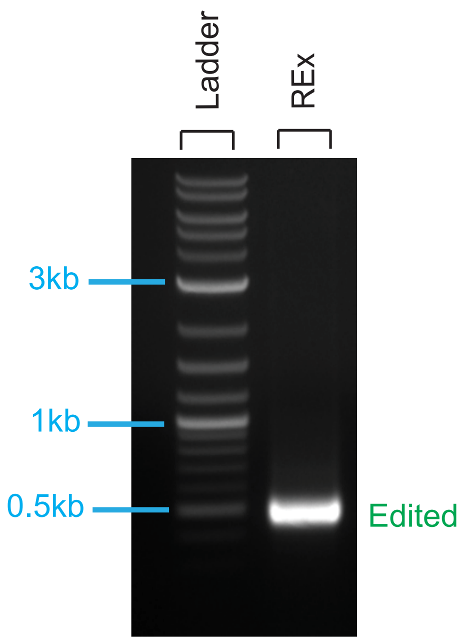

Edited allele expected band size = 480bp

C

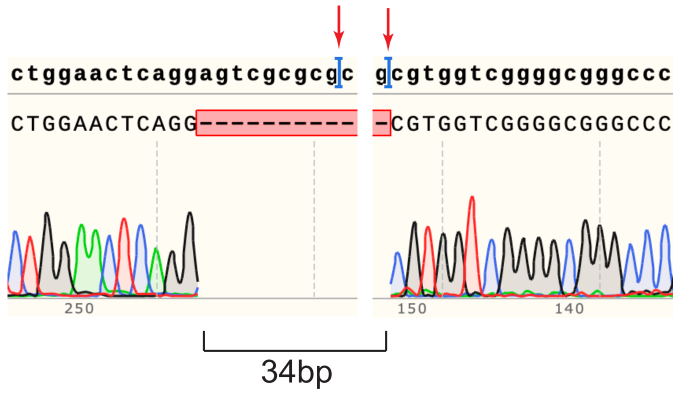

D

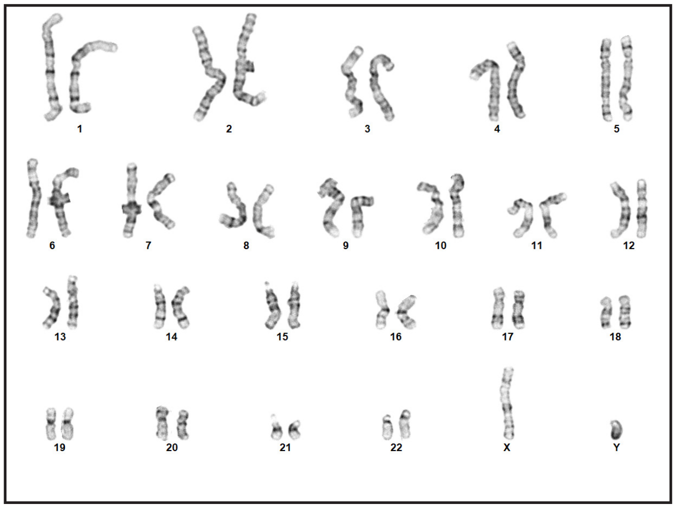

Results: 46,XY
Banding Technique: GTL
Total Counted: 20
Total Analyzed: 7
Total Karyotyped: 2
Band Resolution: Fair
Interpretation: Apparently NORMAL Human Male Karyotype

Supplementary Figure 7. Construction of the WT-REx cell line. (A) Position of the gRNAs (indicated by scissors) and excision primers (purple arrows) used to create and verify, respectively, excision of the repeat region of the *C9orf72* gene in a non-diseased (control) cell line. **(B, C)** Presence of an excision band at the expected band size (B) and Sanger sequencing (C) show the 34 bp excision. We could not perform cut site sequencing because we could not design unique primers inside the GC-rich repeat region. Therefore we relied on clean Sanger sequencing (C) to indicate the line was pure around the cut sites (pink arrows). **(D)** The cell line had a normal karyotype.

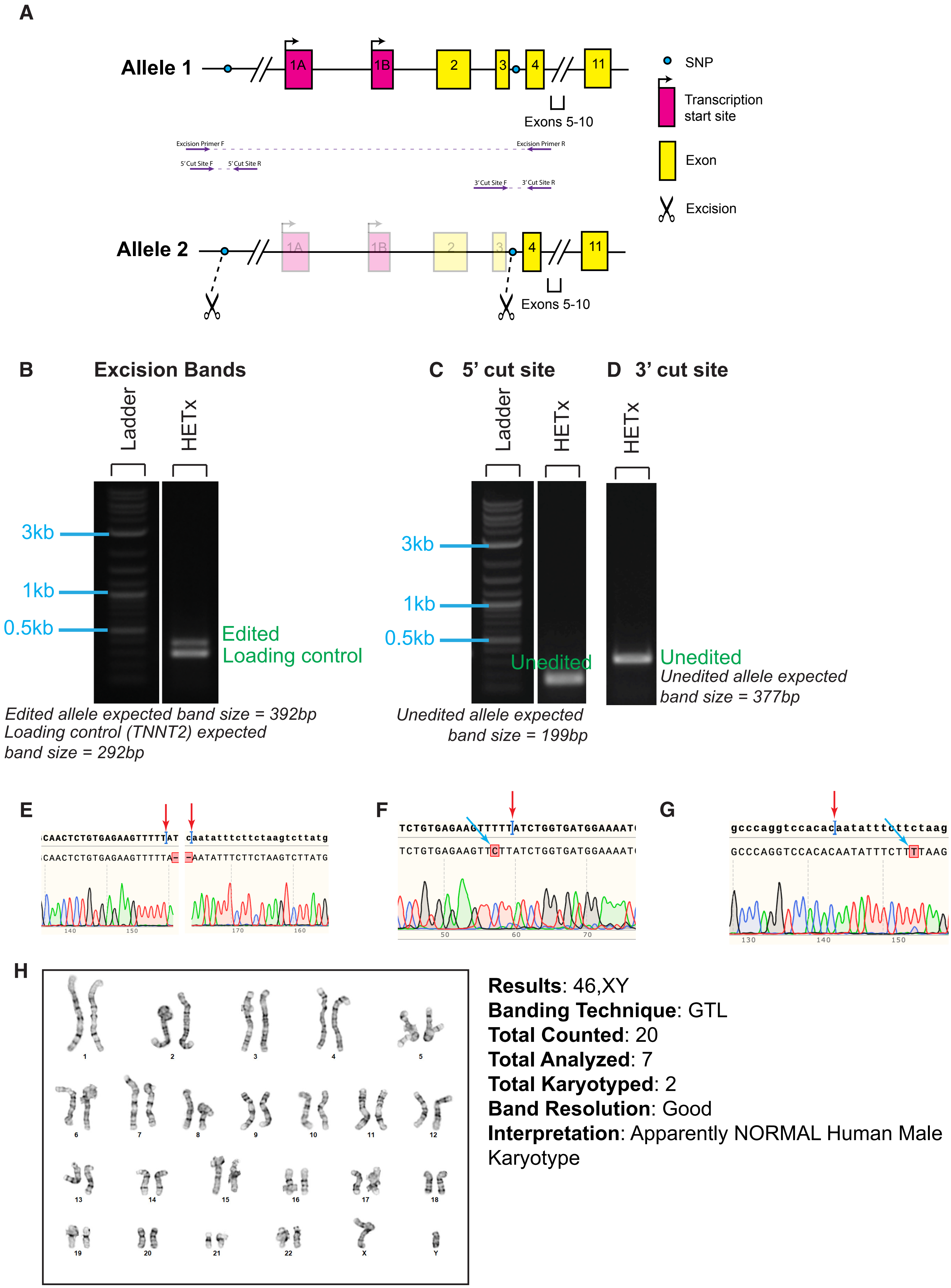
A
Allele 1
1A
1B
2
3
4
11
Exons 5-10
Excision Primer F
Excision Primer R
5' Cut Site F
5' Cut Site R
3' Cut Site F
3' Cut Site R
Allele 2
SNP
Transcription start site
Exon
Excision
B
Excision Bands
Ladder
HETx
3kb
1kb
0.5kb
Edited
Loading control
Edited allele expected band size = 392bp
Loading control (TNNT2) expected band size = 292bp
C
5' cut site
Unedited
Unedited allele expected band size = 199bp
D
3' cut site
Unedited
Unedited allele expected band size = 377bp
E
CAACTCTGTGAGAAGTTTTTAT caatatttcttctaagtcttatg
CAACTCTGTGAGAAGTTTTTA- -AATATTTCTTCTAAGTCTTATG
F
TCTGTGAGAAGTTTTTATCTGGTGATGGAAAAT
TCTGTGAGAAGTTCTTATCTGGTGATGGAAAAT
G
gcccaggtccacacaatatttcttctaag
GCCCAGGTCCACACAATATTTCTTTTAAG
H
Results: 46,XY
Banding Technique: GTL
Total Counted: 20
Total Analyzed: 7
Total Karyotyped: 2
Band Resolution: Good
Interpretation: Apparently NORMAL Human Male Karyotype

Supplementary Figure 8. Construction of the WT-HETx cell line. (A) Position of the gRNAs (indicated by scissors) and excision primers (purple arrows) used to create and verify, respectively, excision of a single allele of the *C9orf72* gene in a non-diseased (control) cell line. SNPs phased to the repeat region (blue dots) were used to target a single allele. **(B-D)** Presence of an excision band (B) at the expected size and preservation of bands at both the 5' (C) and 3' (D) cut sites at the expected sizes indicate the line has a heterozygous excision. **(E-G)** Corresponding clean Sanger sequencing shows the clone is pure and there are no indels on the unedited allele (pink arrow – cut site; blue arrows – SNP). **(H)** The cell line had a normal karyotype.

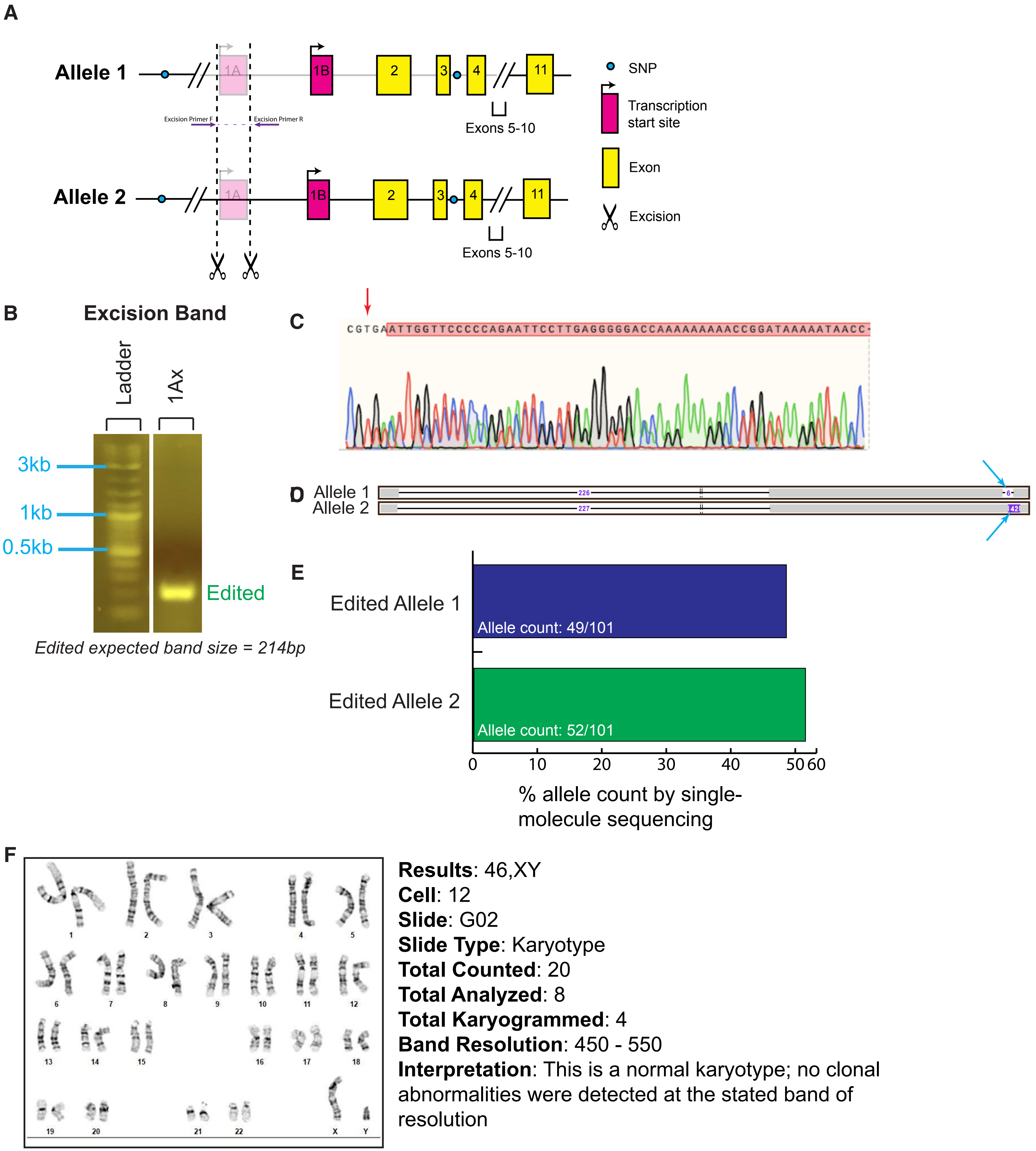
A
Allele 1
1A
1B
2
3
4
11
Excision Primer F
Excision Primer R
Exons 5-10
Allele 2
1A
1B
2
3
4
11
Exons 5-10
SNP
Transcription start site
Exon
Excision
B
Excision Band
Ladder
1Ax
3kb
1kb
0.5kb
Edited
Edited expected band size = 214bp
C
CGTGAATTGGTTCCCCCAGAATTCCTTGAGGGGGACCAAAAAAAAACCGGATAAAAATAACC-
D
Allele 1
Allele 2
226
227
6
42
E
Edited Allele 1
Allele count: 49/101
Edited Allele 2
Allele count: 52/101
0
10
20
30
40
50
60
% allele count by single-molecule sequencing
F
1
2
3
4
5
6
7
8
9
10
11
12
13
14
15
16
17
18
19
20
21
22
X
Y
Results: 46,XY
Cell: 12
Slide: G02
Slide Type: Karyotype
Total Counted: 20
Total Analyzed: 8
Total Karyogrammed: 4
Band Resolution: 450 - 550
Interpretation: This is a normal karyotype; no clonal abnormalities were detected at the stated band of resolution

Supplementary Figure 9. Construction of the WT-1Ax cell line. (A) Position of the gRNAs (indicated by scissors) and excision primers (purple arrows) used to create and verify, respectively, excision of exon 1Ax of the *C9orf72* gene in a non-diseased (control) cell line. **(B)** Excision band was present by PCR at the expected band size. **(C)** Because of messy Sanger sequencing after subcloning which appeared to show a 1 bp overlap in traces after the cut site (pink arrow), we could not resolve whether the clone remained impure or whether there were different editing outcomes on the two alleles. We therefore turned to single-molecule sequencing **(D)**, which indicated a pure clone with a 226 bp excision on one allele and a 227 bp excision on the other using the difference in the repeat number (blue arrow) to differentiate the alleles. **(E)** The percentage of each allele detected by single-molecule sequencing was roughly equal. **(F)** The cell line had a normal karyotype.

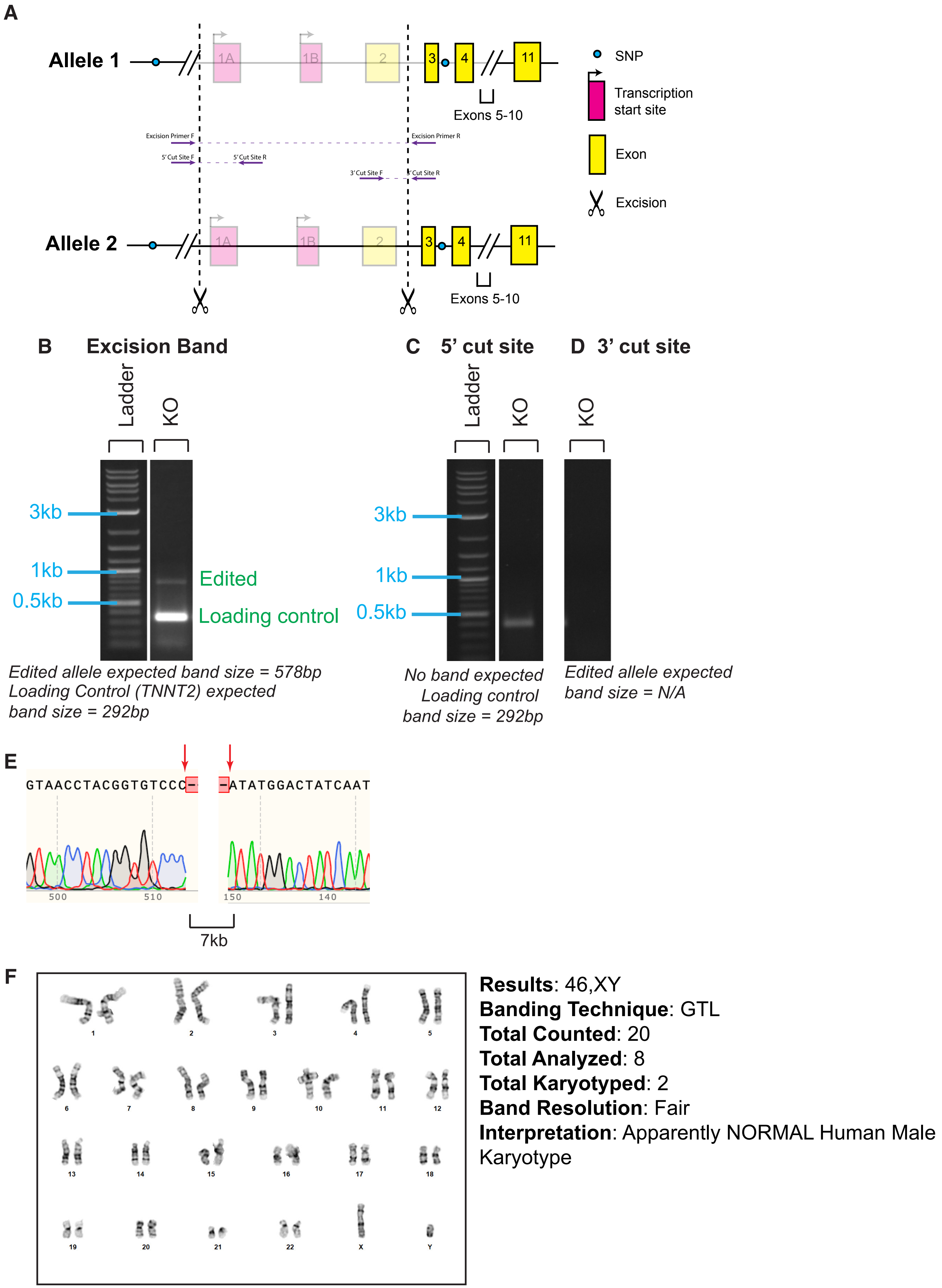
A
Allele 1
1A
1B
2
3
4
11
Exons 5-10
SNP
Transcription start site
Exon
Excision
Excision Primer F
Excision Primer R
5' Cut Site F
5' Cut Site R
3' Cut Site F
3' Cut Site R
Allele 2
1A
1B
2
3
4
11
Exons 5-10
B Excision Band
Ladder
KO
3kb
1kb
0.5kb
Edited
Loading control
Edited allele expected band size = 578bp
Loading Control (TNNT2) expected band size = 292bp
C 5' cut site
Ladder
KO
3kb
1kb
0.5kb
No band expected
Loading control band size = 292bp
D 3' cut site
KO
Edited allele expected band size = N/A
E
GTAACCTACGGTGTCCC-
-ATATGGACTATCAAT
500
510
150
140
7kb
F
1
2
3
4
5
6
7
8
9
10
11
12
13
14
15
16
17
18
19
20
21
22
X
Y
Results: 46,XY
Banding Technique: GTL
Total Counted: 20
Total Analyzed: 8
Total Karyotyped: 2
Band Resolution: Fair
Interpretation: Apparently NORMAL Human Male Karyotype

Supplementary Figure 10. Construction of the WT-KO cell line. (A) Position of the gRNAs (indicated by scissors) and excision primers (purple arrows) used to create and verify, respectively, a 7 kb biallelic excision of the *C9orf72* gene in a non-diseased (control) cell line. **(B)** Excision band was 153 bp larger than the expected 578 bp, corresponding to a smaller than predicted excision. **(C, D)** Absence of WT bands at 5' and 3' cut sites indicate homozygosity. We sequenced the unexpected 400 bp band at the 5' cut site (C) which had no homology to the *C9orf72* locus, indicating a primer off-target. **(E)** Sanger sequencing shows the excision cut sites (pink arrows). **(F)** The cell line had a normal karyotype.

	CRISPOR				
	Score	Genomic Loci	Forward Primer	Reverse Primer	Off-target?
**5' REx**	0.22	chr2_111096413	TCTTTGGGGAACAGGAAACGAA	TTTCCCACTGCCACCATACC	No
	0.22	chr2_110790151	ACAGCGGGTAGGGCTTTAAC	GTGTAGCCAGGACAGGTCAC	No
	0.21	chr11_1436220	AGAGCAAAGGCACCCTACCA	GAGGTATCTCTGCAGAAGGCTG	No
	0.09	chr8_144512745	GTGAGGAACCTGGGGTAAGC	CTGCCGGAAAGCATGTCAGA	No
	0.07	chr2_178351942	CCAAAAGATGCCACCAAGAAACT	TGGAATGGAGTGCCCATGTA	No
**3' Rex**	1.00	chr5_135219436	AAAGCGTCCTCAGTTCCCTG	CCTCTGACCTGGTTACGCTG	No
	0.43	chr4_582136	TGATCCAGCACTGTCCCAAC	ACACTGCAAACCCACCAGAA	No
	0.42	chr1_3453044	CCCGCAGATAGAGGTGTGC	CCCTCTTTCAGTAGCTGCCTC	No
	0.36	chr1_37499731	TTTCCACCTTTGCTCACCACT	CGGGGACTTTTTCCCATTACT	No
	0.33	chr3_98241588	GTGCGCCCACTGTTAAATTCT	TAGGCAGGAACCCACTTTGC	No
**5' 1Ax**	0.50	chr2_207357539	TTGGCTACTTCTGGCGATGG	GAGGGCCAGAACTTGGGAAT	No
	0.49	chr21_41089454	ACCAGTAATGTTGTCCTAAACCTT	GGGAGGTGGGAGTTCAATGG	No
	0.43	chr7_123829356	TGCAGCAATCTATCCGAGGG	AAGCTCCCTGAGAGCAGGTAT	No
	0.42	chr7_5162877	GGTCATGGTGGTGCATACCG	CACACTGTCCTGATTACCGTAGC	No
	0.37	chr5_164953420	TCTGCTCATTGCCAATCCCT	CTGTAGGTGCCAGAGACTTCAA	No
**3' 1Ax**	0.57	chr14_37170608	ACTCAACAAAACAGCATAAGTAGGA	TGGTCAAATTCCGCAATGCC	No
	0.51	chr5_152072966	AGGAGGCGTAGGGTATGTGA	TGAGTAGCACTGGGCCTCTA	No
	0.47	chr11_19359539	TGCGTGTGGGGATTCATAACT	CAGAGAAGTGACCCGACAGC	No
	0.47	chr1_200298374	CCTCAAGAGAGCCTGTAAGACC	AGCCCTGATCATGTTATCGCA	No
	0.39	chr20_24228699	TCGGGACCTAACCTCAAGAATG	CAAGTAGTGGGGGAAAGGAGG	No
**5' HET(Mut)x**	0.59	chr12_92375326	TTGGGGACTTGGACAGGAAGA	ACTCAAAGCCACTGAAAGCAG	No
	0.48	chr18_48748719	AAGGCACCTCCCGAAGATCA	CCTCTGCTGTCTCACTCACC	No
	0.39	chrX_26137636	ACATTTGTGCTACCTCAAGACTTT	CTCTGCTGACTTGAGGCCAT	No
	0.39	chr1_33184187	ACTGACGTGCACCCCTAATG	TCTCACTCGGCTGCAAGAAC	No
	0.33	chr9_89852991	CAAGGGAGAAATGCCAACGC	GATGCCTCTGTCCATTCGCT	No
**3' HET(Mut)x**	0.73	chr12_114544485	GGTGTGCTGCTGCTTTCCTA	TGCCCAATACTGTCCTTCCG	No
	0.66	chr13_25276844	ATGCAAAACAATCACACCGCT	TTCACGGATTTCCAGTGTGGG	No
	0.63	chr1_60919550	ACCTTCACTGATAGCCCCTTTC	CCAGCTGTTGGCTTCTCTTC	No
	0.59	chr13_101497277	TGGAAAGCCTGGGATGTTGT	ACTTGGGGAGTAAGGACCGA	No
	0.58	chr10_73170784	CCCTTCGCACTTTTACTTGGG	ACAGAATCATCTTTTGGGCCTT	No

	CrispRGold				
	Score	Genomic Loci	Forward Primer	Reverse Primer	Off-target?
**5' REx**	None ≤11	N/A	N/A	N/A	N/A
**3' Rex**	None ≤11	N/A	N/A	N/A	N/A
**5' 1Ax**	None ≤11	N/A	N/A	N/A	N/A
**3' 1Ax**	9	chr12:130643855-130643875	GGGACAGGTCCCCCACTTTA	AGGCCCCTGAATCCACAGTA	No
	9	chr11:124539924-124539946	GTGGTGTGGTCATCCCAGTTA	GCCGCTCCCAACTAATTCTCA	No
	7	chr11:19359541-19359562	TGCGTGTGGGGATTCATAACT	ACCTGCTGAACCAAGACAGG	No
**5' HET(Mut)x**	11	chrX:26137638-26137659	CTTACGCGAGGGGCATAAGT	ATGCCCTCAGTCCTCACTCA	No
	11	chr1:24019032-24019053	GAGGTGGCCACGAGGATAAC	GGAGTACCCTCCCGAGAAGT	No
	11	chr1:33184189-33184210	GCCCATTTCCAGGAGTGAGT	TGATCTCTAGTGGACCAGCCA	No
	10	chr12:92375328-92375349	AGTGATGGGGCCTGATAGTCT	GGTTTGGGAGCTAGGTCAGC	No
	5	chr9:27586156-27586177	TGGGCACCTTGGTCCTTTAAT	CAGGAACCAAGCAGCCATGA	No
**3' HET(Mut)x**	11	chr8:109080384-109080404	ACTCTGCATGATGTCAGTCC	GGGAACGTTTTGTGTGGGAA	No
	11	chr14:46240509-46240530	TCTACAGGATGGT	TCTAGAGTGCTGTGGATATGGT	No
	11	chr13:101497278-101497299	AGGATCTGTTTCACAGGGGC	TACGGACTGTGGGGTGAGTA	No
	11	chrX:147236570-147236591	AAGGAGCAACTGAAGGGAAGT	TATTTGGTGGCCATGGGTGT	No
	11	chr11:66759457-66759478	CAGTCCTGGTGTTGATTGTGG	AACCGTACCAACTCTGGCATA	No
	10	chr3:119317892-119317913	AGGCAGAATTGAAGGGCTGAT	ACCGTGAACAAACTGACAGG	No
	10	chr2:160815411-160815432	TGGCTTCCTGCCCATGAAAT	TCCAGTCACTTCAGAACACTCC	No
	10	chr14:84945589-84945610	TGCCCAGACGGTTCTAGGAT	CCACTAAGGGCAAGACCTCAA	No
	10	chr14:81528944-81528965	CAGCCTGCTTTGGTGTTGTC	TGCCTAATGTTATGGGCGCT	No
	10	chr17:55694279-55694299	TCCCTGGCAATGAATGGACAA	GTCACAGTGCTACCGCTCAT	No
	10	chr10:73170786-73170807	CTAGGCTGTCTTCTAGCCACG	GTTGCAACTGAGCCAAGATGG	No
	10	chr13:94563263-94563283	TGCACCCAACCTAGATGGC	CCAGGAGTTAGGCTGGTCTCT	No
	9	chr11:101038966-101038987	AAGCTCTGACCCAAAGGAGC	CAGGGATGGTGAAACCCCTT	No
	8	chr12:114544487-114544508	CTCAGCACATTCTCAGCCCA	AGAAGACACGGGGTTTCACC	No
	4	chr2:195033303-195033324	AGCCACTACAAAGCTGAATGC	TTGCTGGCAACTTCAAACATT	No
	4	chr9:27565102-27565123	CCAGACTTTCCCAAGTGGGTT	GGATTTGGACACGTGGCAATG	No

Supplementary Figure 11. No detectable off-targets in clonal lines. We measured the top 5 predicted off-targets from CRISPOR (https://crispor.tefor.net/) and the predicted off-targets with a score of 11 and below from CrispRGold (https://crisprgold.mdc-berlin.de/). CRISPOR's CDF score predicts the interaction between guide and on-target sequence with 1.0 indicating the strongest and 0 indicating the weakest interaction; therefore, the higher the score the lower the off-target effect in the genome. CrispRGold gives a specificity score of the sgRNA based on the top 3 off-target sites. sgRNAs with a specificity score greater than or equal 12 are unlikely to be off-targets. We designed PCR primers to each of the potential predicted off-targets and sequenced across the parental (unedited) and edited clonal line. We found no differences in sequencing between the two lines (*i.e.,* no off-targets) across all lines and genomic loci tested.

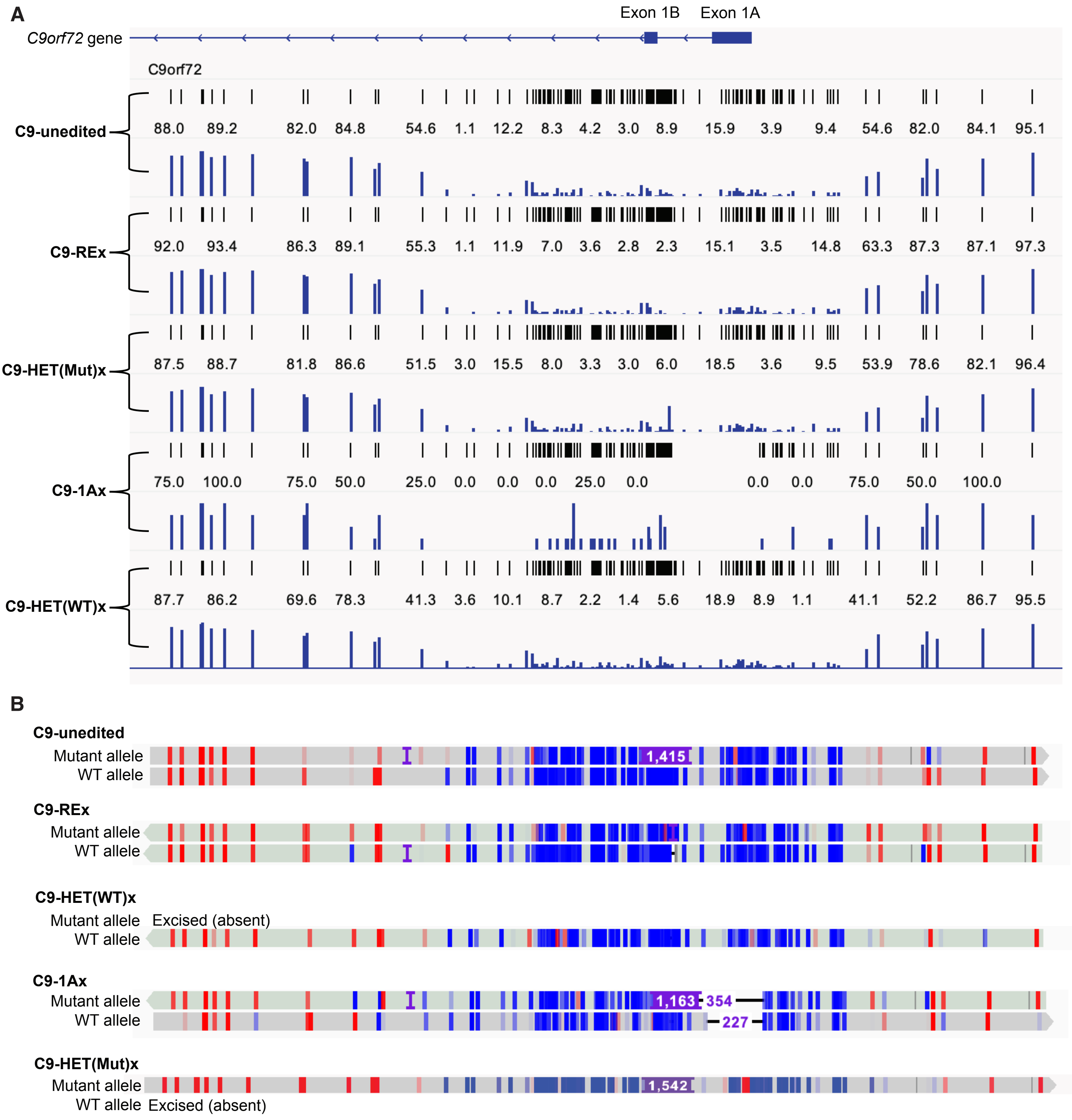

A
C9orf72 gene
Exon 1B
Exon 1A
C9orf72
C9-unedited
88.0 89.2 82.0 84.8 54.6 1.1 12.2 8.3 4.2 3.0 8.9 15.9 3.9 9.4 54.6 82.0 84.1 95.1
C9-REx
92.0 93.4 86.3 89.1 55.3 1.1 11.9 7.0 3.6 2.8 2.3 15.1 3.5 14.8 63.3 87.3 87.1 97.3
C9-HET(Mut)x
87.5 88.7 81.8 86.6 51.5 3.0 15.5 8.0 3.3 3.0 6.0 18.5 3.6 9.5 53.9 78.6 82.1 96.4
C9-1Ax
75.0 100.0 75.0 50.0 25.0 0.0 0.0 0.0 25.0 0.0 0.0 0.0 75.0 50.0 100.0
C9-HET(WT)x
87.7 86.2 69.6 78.3 41.3 3.6 10.1 8.7 2.2 1.4 5.6 18.9 8.9 1.1 41.1 52.2 86.7 95.5
B
C9-unedited
Mutant allele
1,415
WT allele
C9-REx
Mutant allele
WT allele
C9-HET(WT)x
Mutant allele Excised (absent)
WT allele
C9-1Ax
Mutant allele
1,163 354
WT allele
227
C9-HET(Mut)x
Mutant allele
1,542
WT allele Excised (absent)

Supplemental Figure 12. CRISPR editing does not change the methylation pattern in iPSCs. Methylation was determined by single-molecule PacBio sequencing. There were no differences in methylation pattern in a 5-kb region around the repeat expansion in any of the C9-edited cell lines compared to C9-unedited. **(A)** Visualization depicting 5mC (methylation) pattern of a 5 kb region of the *C9rof72* gene centered around the repeat region between exons 1A and 1B for each C9 iSPC line. Each blue bar is the proportion of reads that were predicted to have a 5mC modification probability greater than 0.5, for a given locus. A higher bar indicates consistent methylation at that site across multiple reads for each iPSC cell line. Lower bars (such as in the center of the gene) indicate low probability of methylation. The gene runs from right (5') to left (3'). **(B)** Because all of the reads from both alleles are collapsed into a single file in (A), we also show examples of individual alleles sequenced for each iPSC line. Red signifies a greater predicted probability of methylation at the indicated nucleotide position. Blue signifies lower predicted probability of methylation. The repeat expansion is depicted in purple. Excisions within the sequenced region are depicted by white breaks in sequencing. These data suggest both that CRISPR does not alter methylation and that the changes in pathology and electrophysiology seen after CRISPR editing were unlikely to be due to methylation changes.

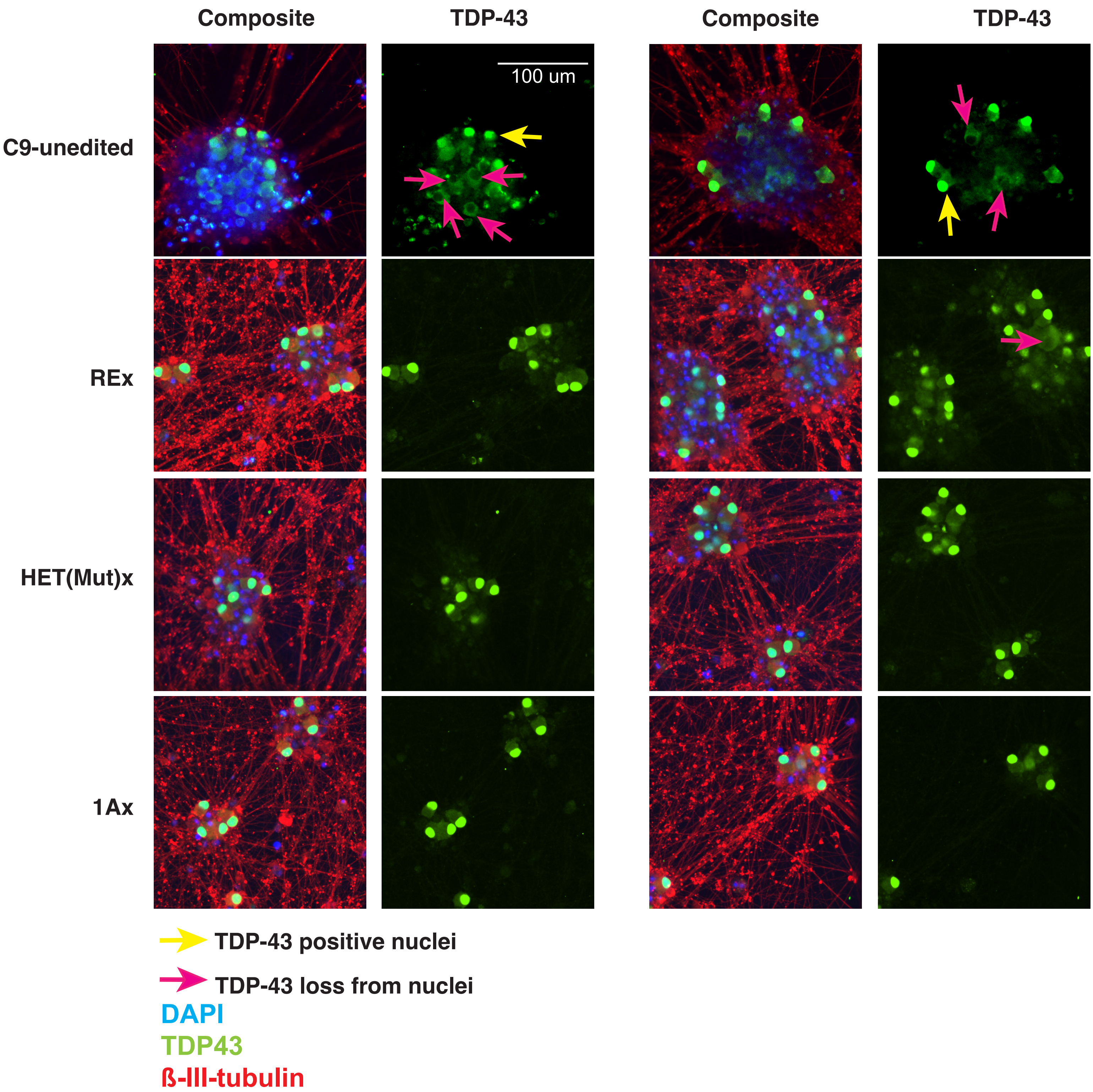
Composite
TDP-43
Composite
TDP-43
100 um
C9-unedited
REx
HET(Mut)x
1Ax
TDP-43 positive nuclei
TDP-43 loss from nuclei
DAPI
TDP43
ß-III-tubulin

Supplemental Figure 13. **Additional TDP-43 images** (supplement to Figure 4 main text). Immunofluorescent images of neurons derived from unedited and edited C9 iPSCs. The neurons were grown for 7 weeks and stained for TDP-43 (green), DAPI (blue) and beta-III-tubulin (red). Two additional example images of each condition are shown. Yellow arrow points to a nucleus harboring TDP-43 and pink arrow to a TDP-43-positive cell whose nucleus is devoid of TDP-43.
